# Rational design based on multi-monomer simultaneous docking for epitope imprinting of SARS-CoV-2 spike protein

**DOI:** 10.1038/s41598-024-73114-3

**Published:** 2024-10-04

**Authors:** Soumya Rajpal, Alex D. Batista, Rüdiger Groß, Jan Münch, Boris Mizaikoff, Prashant Mishra

**Affiliations:** 1 Institute of Analytical and Bioanalytical Chemistry (IABC), Ulm University, Albert-Einstein-Allee 11, 89081 Ulm, Germany; 2 Institute of Molecular Virology (IMV), Ulm University Medical Center, Meyerhofstraße 1, 89081 Ulm, Germany; 3 Department of Biochemical Engineering and Biotechnology, Indian Institute of Technology Delhi, New Delhi, 110016 India; 4 Hahn-Schickard, Sedanstraße 14, 89077 Ulm, Germany

**Keywords:** Bioinspired materials, Polymer chemistry

## Abstract

Among biomimetic strategies shaping engineering designs, molecularly imprinted polymer (MIP) technology stands out, involving chemically synthesised receptors emulating natural antigen-antibody interactions. These versatile ‘designer polymers’ with remarkable stability and low cost, are pivotal for *in vitro* diagnostics. Amid the recent global health crisis, we probed MIPs’ potential to capture SARS-CoV-2 virions. Large biotemplates complicate MIP design, influencing generated binding site specificity. To precisely structure recognition sites within polymers, we innovated an epitope imprinting method supplemented by *in silico* polymerization component screening. A viral surface Spike protein informed epitope selection was targeted for MIP development. A novel multi-monomer docking approach (MMSD) was employed to simulate classical receptor-ligand interactions, mimicking binding reinforcement across multiple amino acids. Around 40 monomer combinations were docked to the epitope sequence and top performers experimentally validated via rapid fluorescence binding assays. Notably, high imprinting factor polymers correlated with MMSD predictions, promising rational MIP design applicable to diverse viral pathologies.

**Supplementary Information:**

The online version contains supplementary material available at 10.1038/s41598-024-73114-3.

## Introduction

The COVID-19 pandemic caused by SARS-CoV-2 has affected millions of lives worldwide^1, ^necessitating further research to enable more effective strategies for managing spread of disease. Existing rapid test kits, drugs approved and in development, and millions of vaccine doses have achieved great successes, but are constrained by the emergence of new variants associated with changes in neutralisation susceptibility and pathogenesis. In such situations, research efforts must adapt to the altering disease dynamics. The development of diagnostics and therapies against pathogenic infections can be fundamentally guided by a comprehension of the host-guest interaction mechanism. When a virus infects a host, the viral components—in particular, the surface proteins—trigger a series of immune responses, including production of antibodies that recognise and neutralise the foreign antigen. For SARS-CoV-2 specifically, the viral Spike protein mediates entry into the host cell by binding to the ACE2 receptor^2,3^. In response to an infection, the immune system produces antibodies that neutralize Spike protein and thus interfere with this binding mechanism. This antibody activity has been exploited to develop *in vitro* assays and biosensors to capture the virus^3–7^. Antibodies exhibit a unique and high-affinity binding capacity thus, their utilisation in therapeutics and diagnostics has become a 'gold-standard’ for majority of diseases.

The development time and expense of antibody-based technologies pose significant challenges and the instability of antibodies further limits their application particularly in low-resource settings^8,9^. Alternatively, antibody mimics such as molecularly imprinted polymers (MIPs) are able to address these drawbacks and can be engineered with selective recognition properties^10^. MIPs are reliable for affordable diagnostics development since they can be chemically synthesized at a fraction of price of antibodies and tailor-made for any target molecule^11–21^ including biologicals like proteins^22–30^ and viruses^31–36^. The standard synthesis process comprises a set of functional monomers and cross linkers to induce polymer formation around the template. Next, the template is extracted using the suitable solubilization agents, leaving behind cavities that are complementary to the template in terms of size, shape and functionality. A template for MIPs against a given pathogen can be the entire virus/cell or a biomolecule unique to this biological species. The latter can serve as an indirect yet precise method of detection, whereas the entire virus may pose a risk during handling^31,35^. Therefore, in this study we selected SARS-CoV-2 Spike protein to highlight the potential of MIPs in diagnostics development. Spike protein is a trimeric glycoprotein with each unit approx. 180 kDa in size and specific expression at the coronavirus surface^2^. The need to template macromolecules (> 1500 Da) prompted the use of advanced methodologies such as surface imprinting in lieu of conventional bulk imprinting procedures to prevent erroneous cavities within the polymer and template entrapment. Surface imprinting involves the immobilization of the template on a surface to enable uniform imprinting and easy extraction through fast mass transfer of template molecules^12,26,37–40^.

Biomacromolecular interactions essentially converge at limited regions exemplified as ‘epitope’ and ‘paratope’ within an antigen-antibody complex. Drawing from the mechanistic insights offered by biological complexes, we sought to decrease the size of the template in order to optimise the binding efficacy of MIPs or artificial antibodies. We have selected a short sequence of amino acids (‘epitope’) derived from the receptor binding domain of Spike protein and used it in a unique epitope imprinting strategy. This strategy served a dual purpose – firstly, the development of monoclonal-type synthetic antibodies was enabled that target a single epitope. Secondly, by reduction of the template dimension an improved fidelity of the binding sites, and thus, selectivity of the resulting MIPs was anticipated^41–44^.

Polymers exhibit an inherent adsorption trait that creates the possibility of non-specific molecular interactions, which may potentially limit the performance of MIPs^10^. Improvements in MIP specificity are possible with the application of computational predictive design^45^. For macromolecular complexes involving higher number of atoms, computational methods have been inspired from traditional drug design approach^23,46,47^. In general, monomers are docked to the template/epitope to evaluate their binding affinity and those mimicking the multi-point interaction observed in the antigen-antibody complex are considered most-suitable for imprinting. This is based on a well-established correlation of binding affinity among the pre-polymerization components with the MIP performance. Ideally, higher strength and diversity of interactions predicted between monomer and template should translate into improved experimental binding of resulting MIP and target^48–52^.

This can be achieved using a distinct class of silane monomers that facilitate the incorporation of diverse functional groups into a polysiloxane network and exhibit compatibility with hydrophilic environments (required by biotemplates)^27,53,54^. Combined use of multiple monomers has shown to be an effective strategy to enhance the specificity of MIP binding sites; this concept essentially mimics the antibody ‘paratope’, which integrates unique functionalities from multiple amino acids^55,56^. For example, Bhakta et al. used four silane monomers with unique functionalities to efficiently bind with template proteins^27^. In order to *in silico *replicate the experimental procedure of combining multiple monomers, the conventional approach of single monomer docking, which estimates the binding affinity of monomers docked individually, may be inadequate. As a result, we have developed a novel multi-monomer combinatorial screening to examine how different monomers can interact with the template ‘collectively’. The proposed approach entails the establishment of a tight parallelism between the experimental design and the objective of enhancing the binding specificity of MIPs.

**Fig. 1:**
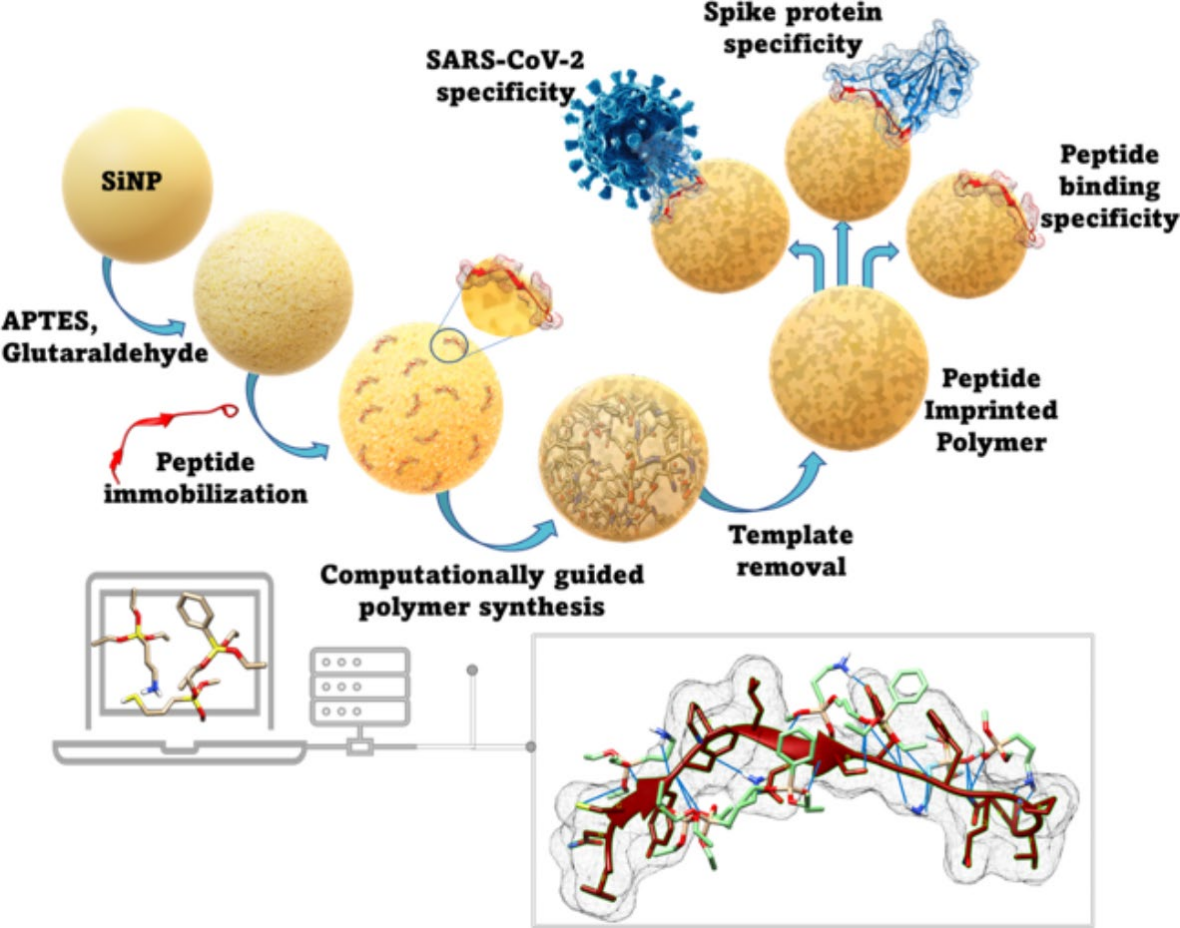
Schematic representation of design strategy for MIPs specific to epitope (peptide) selected from Spike protein of SARS-CoV-2. Silica nanoparticles (SiNP) were employed for surface imprinting of the target peptide (red). Computationally screened monomer combinations are used to synthesize MIPs. Following template removal, the core-shell type nanoparticle-MIP system so-formed is specific for binding the peptide as well as the virions. The box represents the modeling for multipoint interactions between the monomers (green) and the peptide (red). The blue lines mark the intermolecular H-bonding interactions.

The present study aimed at comprehensively harnessing the advancements in protein imprinting including surface imprinting, epitope imprinting, combinatorial synthesis/screening, computational modeling while adding a novel MMSD (multi-monomer simultaneous docking) approach to the latter. In this study, a library of 11 commonly used silane monomers has been combined to screen (36) distinct multi-monomer combinations and investigate the impact of multiple monomer binding with a (bio)template. Based on this, the experimental binding performance of the best predicted monomer combinations were subsequently examined, thereby validating the MMSD computations. The derived MIPs were synthesized in a core-shell format using silica core nanoparticles and the predicted monomer combinations using the epitope derived from Spike protein as the template (Fig. 1). Finally, in a proof-of-concept study, virus-like-particles that contain all structural proteins of SARS-CoV-2 virions including Spike were deployed to evaluate the binding efficacy of MIPs while demonstrating the capacity of epitope-templated MIPs to capture the entire Spike protein and even the entire virus-like-particles (VLP). To make the detection strategy more sensitive, fluorescamine - a fluorescent dye - was utilised for the first time in a biomimetic MIP assay. This dye reacts with the primary amines associated to the N-terminal and/ lysine amino acid of proteins/peptides and forms a fluorescent product that could assay the template concentration in solution up to micromolar levels.

Overall, the present strategy serves as a model approach for essentially any epitope template and can be readily adapted, e.g., for mutated epitopes pertinent to the SARS-CoV-2 virus as well or for other viral diseases. Furthermore, this computational approach offers a readily available framework to rationally design MIPs with any set of monomers, i.e., not limited to the monomer library used in the present study. The MMSD approach provides a crucial starting point for guiding MIP design, significanlty reducing the number of potential monomer combinations before progressing to polymer-level modeling. This enables a thorough analysis of interactions, both within the imprinted cavity and on the polymer surface, particularly in relation to the different functional group arrangements involved.

## Results and discussion

### Rational design of epitope imprinted MIPs for SARS-CoV-2

Spike protein is the major surface glycoprotein of SARS-CoV-2 and is crucial for interaction with the cellular receptor ACE-2 and this infection. Antibodies have also been developed to target this protein for SARS-CoV-2 detection and potential therapeutic interventions. In our study transitioning from natural to artificial antibodies/MIPs, we used a novel “epitope” imprinting approach that can mimic the biological selection process. We have identified a 17 amino acid sequence (F^486^NCYFPLQSYGFQPTNG^502^) from the receptor binding domain (RBD) which encompasses 10 pivotal contact points, intricately involved in interaction with the ACE2 receptor^57^. The peptide sequence and conformation has been extracted from the Spike protein’s crystal structure data (PDB ID: 7JMO) to maintain the native epitope conformation as observed in the protein structure. This specific structure is anticipated to closely mimic the natural antibody developement process within MIPs. The peptide’s sequence predominantly adopts a linear, extended coil conformation, with a specific region (LQSYGF) exhibiting a helix-like arrangement, as confirmed through both structural analysis and molecular dynamics simulations (Fig. 1S). This chosen peptide model serves as a pivotal element in our study, demonstrating versatility for application in addressing other epitopes or mutations.

For enhanced MIP design, we utilised computationally predicted polymer composition and deployed a library of silane monomers, suitably used for biomolecule imprinting^27,58–60^. Several silane monomers (Table 1) having an ability to offer diverse functionalities in the mixture during imprinting were individually docked onto the target peptide to analyse the binding affinity with the template. The monomer PTES showed the highest binding energy (-3.13 kcal/mol) with the peptide and most of the contributions were from π-π interactions between the phenyl groups of PTES and aromatic amino acids dominant in peptide sequence. PTES was followed by DIDMS, MTMS, IBTES and TMOS that were majorly involved in π-alkyl and alkyl bond formation with the peptide. Monomers like UPTMS, CETES, APTMS, APTES and MPTMS that were selected for their ability to engage in H-bonding interactions, exhibited comparatively reduced binding affinity with the peptide. This may be attributed to the prevailing hydrophobic character of the peptide. The unexpectedly lower binding score for MPTMS is also noteworthy, especially given the presence of cysteine in the peptide. Table 1 presents the mean binding energy of the highest ranked cluster associated with each monomer, which is further used for comparing and ranking the 11 monomers. Simultaneously, other clusters were employed to characterise the multi-point interactions and establish the utility of the monomer for effective imprint formation. In this way, we have investigated the diverse monomer binding domains on the peptide. Evidently, PTES exhibited substantial number of high affinity and multi-point interactions followed by monomers like UTPMS, APTES, APTMS that display lower affinity but a notably higher count of interaction points on the peptide.

**Table 1:** Single monomer docking: Screening of silane monomers with SARS-CoV-2 specific peptide/epitope.

Monomer	Mean Binding Energy (kcal/mol)
Phenyltriethoxysilane (PTES)	-3.31
Diisobutyldimethoxysilane (DIDMS)	-2.93
Isobutyltriethoxysilane (IBTES)	-2.68
3-(methyltrimethoxysilane) (MTMS)	-2.65
(2-cyanoethyl)triethoxysilane (CETES)	-2.55
Tetraethylorthosilicate (TEOS)	-2.45
Ureidopropyltrimethoxysilane (UPTMS)	-2.39
Trimethoxy(octyl)silane (TMOS)	-2.26
3-Aminopropyltriethoxysilane (APTES)	-2.05
3-Aminopropyltrimethoxysilane (APTMS)	-1.95
(3-Mercaptopropyl)trimethoxysilane(MPTMS)	-1.95

High negative binding energy (kcal/mol) is indicative of higher affinity of binding. Monomers are arranged in ascending order of the obtained binding energy values.

### Design of multi-monomer computational screening

**Fig. 2:**
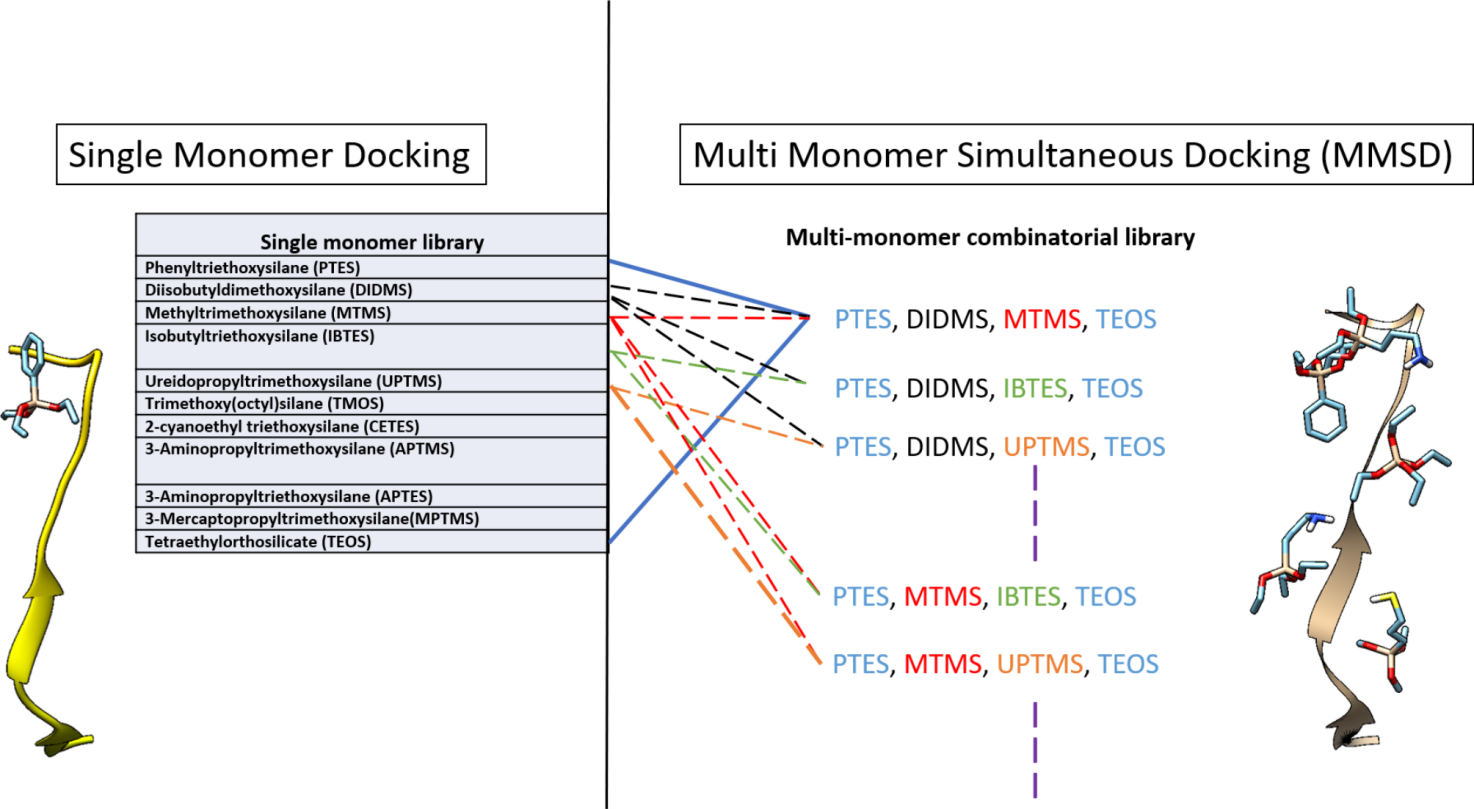
Design of MMSD simulations based on single monomer docking. For MMSD simulations, a library of polymer combinations (PC) was generated. Firstly, each combination was composed of four monomers to introduce optimum amount of functionalities in the polymer combinations. Secondly, PTES and TEOS were kept constant in each PC as PTES was the highest scoring monomer and its phenyl ring interacts extensively with the highly hydrophobic target peptide; and TEOS served as the cross linker. Remaining monomers were used to form suitable monomer combinations and employed for MMSD (Table 1S, SI).

The docking scores for individual monomers can demonstrate their reliability for synthesising precise MIPs, particularly in the case of hydrophobic monomers like PTES and DIDMS. This is attributed to the peptide’s aromatic amino acid composition, which aligns favorably with π-π interactions, further affirming the robustness of these interactions. However, in case of MPTMS, characterised by low binding energy score, it is difficult to anticipate a corresponding decrease in MIP performance. This is particularly noteworthy because the template includes a cysteine residue, which is known to form strong interactions with MPTMS.

An even more significant observation is that this method of docking single monomers at a time, diverges from our intended experimental design approach. Our objective centers on the utilization of a combinatorial screening technique encompassing multiple monomers, thereby introducing a spectrum of diverse functionalities during the experimental synthesis of MIPs. This approach yields MIP cavities that mimic the unique design of antibody paratopes. These MIP cavities expose a variety of functionalities in a configuration that fosters the creation of specific binding sites. In the natural course of antibody development, multiple points of interaction between natural amino acids and the antigen’s epitope lead to the assembly of the antibody binding domain with high affinity. To capture this dynamic, we have introduced a method involving the simultaneous docking of multiple monomers. This technique enables the comprehensive docking of all monomers both individually and in various combinations. This rigourous analysis of the polymerization mixture facilitates the identification of interactions—both high and low affinity—with the peptide.

Within the present library, there exists the capacity to computationally screen in excess of 2000 monomer combinations (without repetition). These combinations can span from 3 to 11 monomers. Most of the studies utilizing silane monomers indicate the use four monomers as optimum for introducing an adequate array of chemical groups into the complex^27,60^. Consequently, we initiated our study with four-monomer combinations to be able to experimentally validate our first-of-its-kind study. This decision of limiting the size of combinations brought down the screening size to up to 330 combinations; still an extensive number for experimental synthesis. Finally, we decided to fix two monomers in each combination, which were PTES, exhibiting the highest binding affinity, and TEOS, that has a well-established function as a cross-linker (Fig. 2). Not only through predicitve analysis, but also inspired from previous literature, phenyl group containing (PTES) monomer is well suited to form strong hydrophobic (π-π) interactions with aromatic amino acids dominated peptide. We also fixed the order in which we added the monomers, for example, TEOS being the cross-linker was added in the end both computationally (in the .dpf file) and experimentally. This design for the MMSD computations resulted in a library of around 36 combinations that were screened against our target peptide (Table 1S, SI). The outcomes included the calculated binding energies for each monomer when docked simultanoeusly and was subsequently extracted for comparative assessment with single monomer docking method. For this, we inspected the variance in binding affinity in individual monomers and their respective combinations.

### Multi-monomer combinatorial screening for predictive MIP design

This study employs a simultaneous docking approach involving four monomers to emphasize the distinct binding patterns that differ from those observed when the monomers are individually present in the solution. It can offer insights into the influence of one monomer’s presence on the other monomer’s binding affinity and points of interaction. Furthermore, an increased number of monomers could potentially contribute to the formation of a robust and stable complex with the target, thereby inducing fewer conformational changes in the template.

First, to generate a comparison of every polymer composition we extracted the mean binding energy scores of each monomer from their first binding cluster and created a sum of all four monomer scores (‘MMSD sum’) (Table 1, SI). Interestingly, the highest sum (-11.61 kcal/mol) was obtained for the polymer combination (PC) containing PTES, APTMS, APTES, TEOS (PC 34). We set a threshold to classify ‘high affinity’ combinations with sum < -11 kcal/mol. Based on this, the resulting 14 combinations were comprised majorly of APTMS (50%) and/ APTES (43%). Previously, these monomers showed notably lower binding affinity with the peptide when subjected to single monomer docking. However, their distinct behavior when combined, resulting in elevated MMSD sums, underscores the enhanced influence achieved through the concurrent application of monomers.

**Fig. 3:**
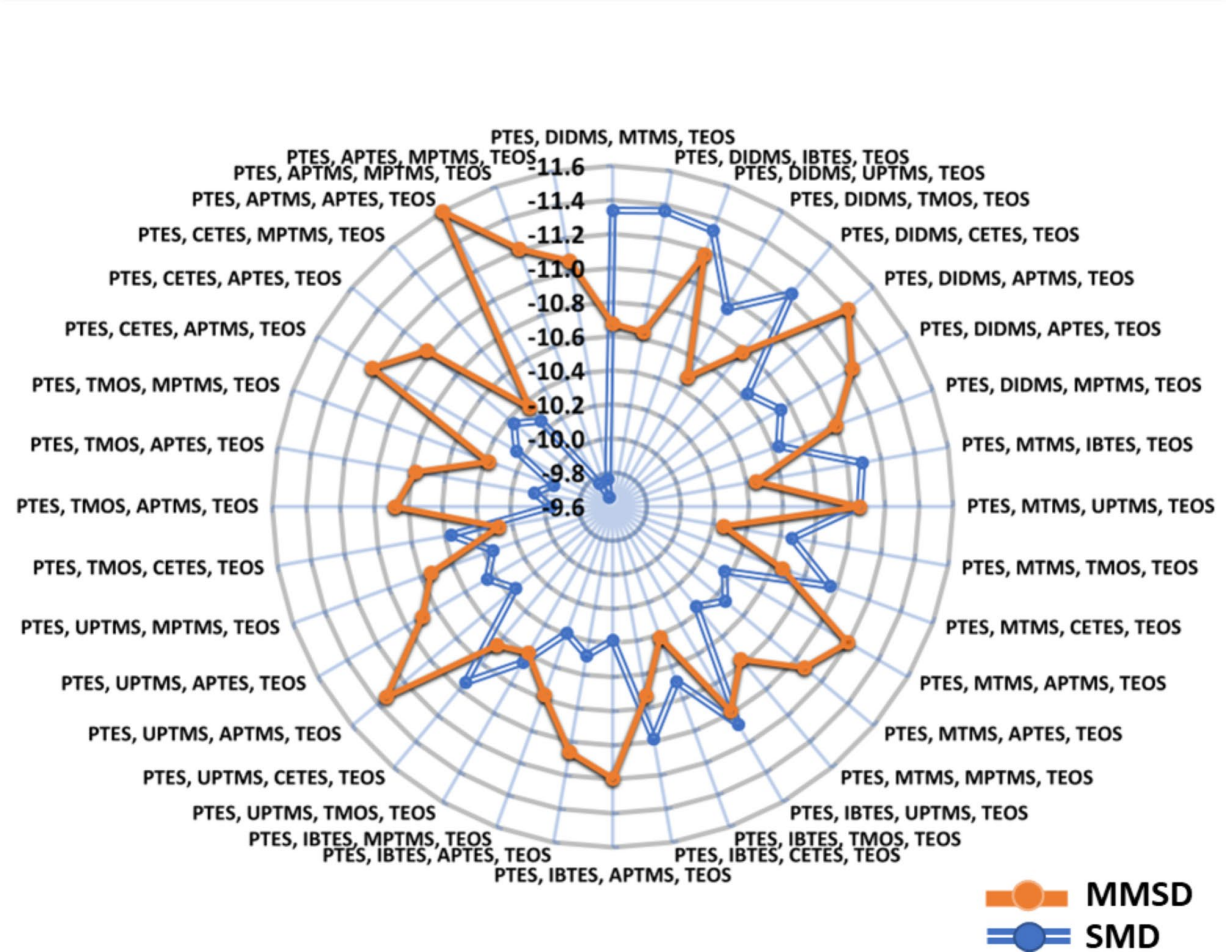
Comparison of MMSD and SMD scores. Representation of the MMSD sum and SMD sum for every polymer combination as orange and blue bullet on every spoke, respectively. Trendline marks the overall change (increase or decrease) contributed by presence of different monomers. The distance from the centre is marked as the binding energy (BE) scale ranging from − 9.6 kcal/mol to -11.6 kcal/mol. Higher negative binding energy values or ∆G represents better binding affinity to the template.

To further understand the evolving trends, binding energy of monomers based on the composition of the 36 combinations was extracted from Table 1 and was cumulated to incorporate as ‘SMD sum’ in Table 1S, SI for thorough comparison. For example, the ‘SMD sum’ for PC 34 was computed as -9.76 kcal/mol where it was rendered as the second lowest performing combination, after PC 35 (-9.66 kcal/mol). In general, the SMD sums spanned from − 9.66 to -11.63 kcal/mol, while the MMSD sums exhibited a narrower range from − 10.26 to -11.6 kcal/mol. This signifies an overall enhancement in binding energies during the course of combinatorial screening (Fig. 3). The stark differences between SMD and MMSD total scores, especially observed in case of PC34 (∆=1.85 kcal/mol) established that simultaneous docking can identify the potential of better performance of a composition with the template molecule. Moreover, it also allows to filter the combinations where there is a decrease in the binding affinity of the monomeric complex as seen in combinations like PC 1, 2 and 3 where MMSD sum has decreased compared to the SMD scores. MMSD can help to capture the characteristics related to the energetic coupling of monomers and also the dynamics and intermediates of multiple monomers binding to the peptide.

### Evaluation of monomer effects

The comparative analysis elucidated in the preceding sections necessitates further elaboration to effectively ascertain polymer combinations characterized by augmented MMSD scores. Subsequently, such combinations can be judiciously prioritized for experimental synthesis. To enhance our rationale, we meticulously examined the impact generated by individual monomers in a way that could establish their significant contribution in a given polymer combination. Therefore, we noted the change in binding affinity of every monomer when applied in SMD compared to when applied in combination. For example, PTES shows a binding energy (BE) value of -3.31 kcal/mol during single monomer docking and in each of the 34 MMSD combination, its BE has either remained same or slightly increased (Table 1S, SI). However, this is not true for DIDMS where binding affinity has mostly decreased from − 2.93 kcal/mol to an average of -2.75 kcal/mol in every involved combination (PC 1 to PC 8). Similarly, for IBTES and MTMS there is change from − 2.7 kcal/mol to roughly − 2.5 kcal/mol in each case. During molecular imprinting, a strong complex is formed by several weak interactions with the template, therefore small differences in the binding energy values can translate significantly to the quality of the templated cavity. This principle adheres to the concept that while non-covalent interactions may be weak individually, they exhibit collective strength through cooperation.

The effect on other monomers when applied in combination is summarised in Table 2 for simpler understanding of the influence on MMSD sums. This monomer-wise analysis can provide the interpretation of the contrasting performance of PC 34. Clearly, all the combinations containing APTMS and APTES have seen a great increase due to their enhanced binding energy values compared to that seen in SMD. Particularly, APTMS has shown a 60% increase from its binding energy of -1.95 kcal/mol in SMD to an average of -3.1 kcal/mol in MMSD combinations and this is followed by APTES and MPTMS with 44% and 30% increase, respectively. The enhancement in binding energy of MPTMS provides a more robust confirmation of the altered and improved dynamics of monomer binding upon its inclusion in multiple monomer combinations. Nonetheless, the question of MPTMS not performing well when docked singly to a cysteine-containing peptide still remains and is subject of a separate and comprehensive study. UPTMS is also one of the monomers that has shown a uniform increase in the binding affinity during MMSD calculations. Conversely, TEOS has demonstrated a notable reduction in the binding energy values (negative ∆G) which may be explained by its non-competitive binding compared to other monomers possessing functional modifications. Given that TEOS serves as a cross-linker and necessitates inclusion in every synthesis, its contribution has been factored into each MMSD calculation. Finally, the monomer-wise investigation has influenced the selection of APTMS, APTES, MPTMS, PTES and UPTMS based combinations which are predicted to yield high-affinity selective MIPs. Additionally, we have also checked combinations that incorporate monomers with high rankings from the SMD approach, such as DIDMS, MTMS, and IBTES.

Concurrently, we examined the non-covalent interactions at the monomer level that were consistently observed with the peptide sequence, as indicated in Table 2S, SI. Firstly, PTES was the only monomer mediating a unique set of π-π interactions. Then, π-alkyl interactions were common for DIDMS, IBTES but also seen with PTES and APTES. Although APTES, APTMS, and UPTMS predominantly engaged in hydrogen bonding interactions, it is noteworthy that the latter, UPTMS, remarkably facilitated the highest density and quantity of H- bonding interactions with nearly all residues. This phenomenon was so pronounced that alternative interactions, aside from hydrogen bonding, were seldom observed. Similarly, sulphur containing MPTMS formed H-bonds and as expected, also with cysteine residues. Specifically, methoxy silane monomers commonly formed a few π-sigma interactions. Overall, the maximum multi-point binding was observed for UPTMS followed by APTES, APTMS, MPTMS, PTES and the least by DIDMS, IBTES and MTMS. Based on the comparison of BE scores and binding interactions, combinatorial screening predicts a superior performance for PCs in an order involving electrostatic interactions/H-bonds with the peptide followed by π-π bonds and others like π -alkyl bonds, alkyl and van der Waals interactions.

**Table 2:** Single monomer docking scores of monomers qualitatively compared with their scores when applied in multi-monomer simultaneous docking.

Monomer (Arranged in ascending order of binding energy values, as obtained from single monomer docking)	Qualitative change in binding energy (BE) values (kcal/mol) in MMSD as compared to single monomer docking	Estimated % change in the binding energy (BE) in MMSD as compared to single monomer docking
Phenyltriethoxysilane (PTES)	Same	-
Diisobutyldimethoxysilane (DIDMS)	Decreases	6%
Isobutyltriethoxysilane (IBTES)	Decreases	5%
3-(methyltrimethoxysilane) (MTMS)	Decreases	7%
(2-cyanoethyl)triethoxysilane (CETES)	Decreases	6%
Tetraethylorthosilicate (TEOS)	Decreases	7%
Ureidopropyltrimethoxysilane (UPTMS)	Increases	16%
Trimethoxy(octyl)silane (TMOS)	Same	-
3-Aminopropyltriethoxysilane (APTES)	Substantially increases	44%
3-Aminopropyltrimethoxysilane (APTMS)	Substantially increases	60%
(3-Mercaptopropyl)trimethoxysilane(MPTMS)	Substantially increases	30%

### Experimental screening based on predictive MIP design

The experimental synthesis of MIPs and NIPs was prompted by the identification of high affinity monomer combinations suggested by the comprehensive analysis and monomer-wise analysis, discussed in previous sections. The maximum improvement in the binding energy scores was seen in the combination involving APTMS, APTES followed by those containing either one of these along with MPTMS and/or UPTMS. We selected only those combinations that showed a uniform increase in the binding energy of the monomers when applied in MMSD (Table 2). As a control, we considered two exemplary compositions (polymer composition (PC) V and VI) involving monomers that showed a general decrease in BE when applied in MMSD, that are DIDMS, IBTES and MTMS. More importantly, to validate the assumption of keeping PTES common in all combinations that was based on its suitability and good performance in SMD and MMSD calculations, we designed a composition with APTMS, APTES but without PTES (PC VIII). Finally, TEOS is additionally used as the cross-linking agent in all these compositions. During synthesis, the monomers were added in the sequence that they were screened and are mentioned in Table 3.

**Table 3:** Eight polymer compositions (PC) tested experimentally, based on rationally selected monomer combinations (Table 1S, SI) derived from computational screening.

Polymer composition	Monomer 1	Monomer 2	Monomer 3	Monomer 4	Monomer combination
PC I	PTES	APTMS	APTES	TEOS	MC 34
PC II	PTES	APTMS	MPTMS	TEOS	MC 35
PC III	PTES	UPTMS	APTES	TEOS	MC 25
PC IV	PTES	UPTMS	APTMS	TEOS	MC 24
PC V	PTES	DIDMS	MTMS	TEOS	MC 1
PC VI	PTES	DIDMS	IBTES	TEOS	MC 2
PC VII	PTES	APTES	MPTMS	TEOS	MC 36
PC VIII (Control)	-----	APTMS	APTES	TEOS	

**Fig. 4:**
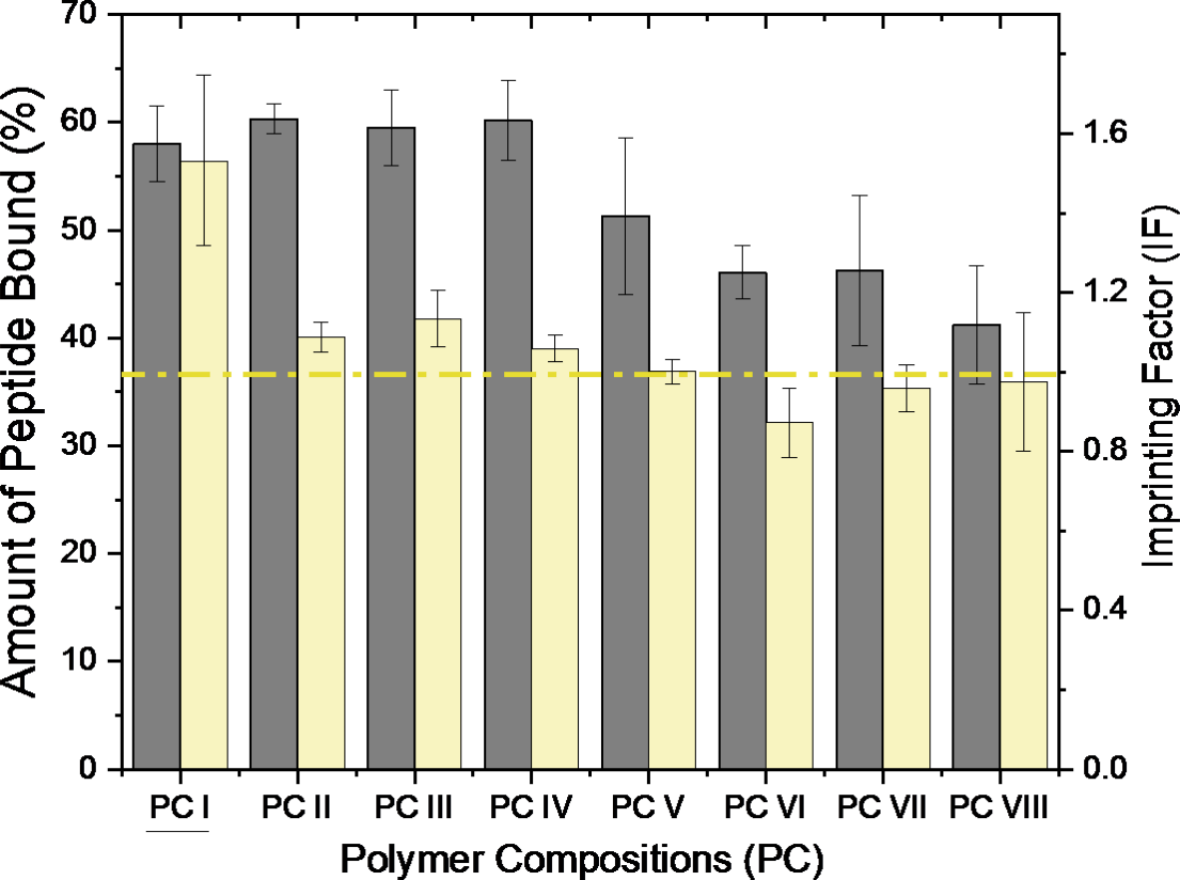
Comparison of performance of PC I to VIII in terms of average binding capacity (BC) and imprinting factor (IF). These compositions were derived from rationally designed and screened monomer combinations through multi-monomer simultaneous docking. PC VIII is a control without PTES. Notably, the low performance of PC VIII reflects on the importance of PTES in each composition. The yellow line indicates an IF of 1, and clearly, PC I exhibits the highest IF (~ 1.5), aligning with its high performance in *in silico* studies. Synthesis and experiments were performed in triplicates. Error bars indicate standard deviation (± SD).

During the assessment of the MIP performance, all eight polymer compositions bound an average of 50% of the peptide from the initial solution (Fig. 4). The maximum reached till 60% in PC IV and the lowest was seen in PC VIII (40%) and clearly, the latter established the importance of PTES in the design. Noteworthy differences were identified in terms of the imprinting factor (IF), as with PC I that showed the maximum value of ~ 1.5. Furthermore, it is unequivocal that π-π interactions serve as additional bonds that positively influence the binding capacity (BC) of all compositions, except PCVIII. Next, PC V and VI involving DIDMS, MTMS, IBTES translated to comparatively poor performances in terms of both, BC and IF. In fact, PC VI was the composition with the lowest IF. This observation can be deduced from the interaction analysis, where PC VI appears to be primarily governed by hydrophobic interactions, which are notably less robust than H- bonds. This could potentially have hindered the accurate templating of the peptide structure. Among these, PC V includes MTMS that allowed more H-bonding (Table 2S, SI) and so, can support its better performance than PC VI.

The remaining compositions involving APTES and APTMS are marked by amino-group based electrostatic interactions enabling improved MIP performance in terms of BC. Slight increase is seen where one of the monomers is APTMS (PC II and PC IV) compared to those containing APTES (PC III and PC VII) however, this will need further validation and will depend on the differential hydrolysis rate of these monomers along with the interference from remaining monomers in combination. Since the peptide contains a cysteine group, it was expected that the MIPs containing MPTMS shall be templated uniquely and more specifically, but this was observed only slightly in one of the two comprised combinations (PC II compared to PC VII). Similarly, the combinations containing UPTMS didn’t result in high imprinting factors. Interestingly, even though UPTMS showed slight increase in BE during the MMSD simulations compared to SMD, the BC of the MIPs and NIPs of composition PC III and PC IV greatly increased. This observation can be attributed to the substantial capacity of UPTMS to form H-bonding with nearly all the amino acid residues (Table 2S, SI) that may have resulted in increased non-specific binding through NIPs.

Overall, the experimental outcomes align with the results obtained from computational screening. This provides valuable insights into the diversity of interactions in the complex that menaningfuly translated into the polymer performance. Furthermore, the binding energy scores derived from SMD are not entirely conclusive, particularly in cases where monomers are employed in combination. Evidently, if experimental synthesis was inspired from SMD, then the best performing compositions would comprise of DIDMS, IBTES, MTMS which was clearly not the case. We have introduced MMSD method that cumulatively accounts for multi-point interactions with the template and also, analyses the effect of one monomer on other. The choice of monomers definitely depends on the template but ensuring stronger H-bonding interactions enhances the MIP binding capacity. In this case, as the peptide contained aromatic residues, ensuring other hydrophobic bonds (even though weak) like π-π interactions uniquely contributes to pave the way for improving MIPs. It is also noteworthy to avoid an excessive density of interactions, engaging all types of amino acid residues alike as it can directly impact the IF. Therefore, the emphasis on diversity and distinctiveness in interactions becomes crucial. Take for instance, PC I, which demonstrates optimal performance, exhibits hydrogen bonding at specific points alongside a combination of hydrophobic interactions and van der Waals interactions at others. However, to gain clarity of the exact mechanism, potential molecular dynamics studies can further highlight the set of monomeric interactions over time.

### Specificity analysis

Imprinting factor alone cannot be considered a determinant of the specificity. Higher binding NIPs characterize the good quality and therefore the binding capacity of the material^58^. Simultaneously, the MIPs characterise the specific structuring of the cavities. In this direction, PC I shows highest performance in terms of BC and the highest IF, however for a definitive characterization of specificty, the IF calculation should be complemented with the binding assesmmnet involving non-specifc proteins.

**Fig. 5:**
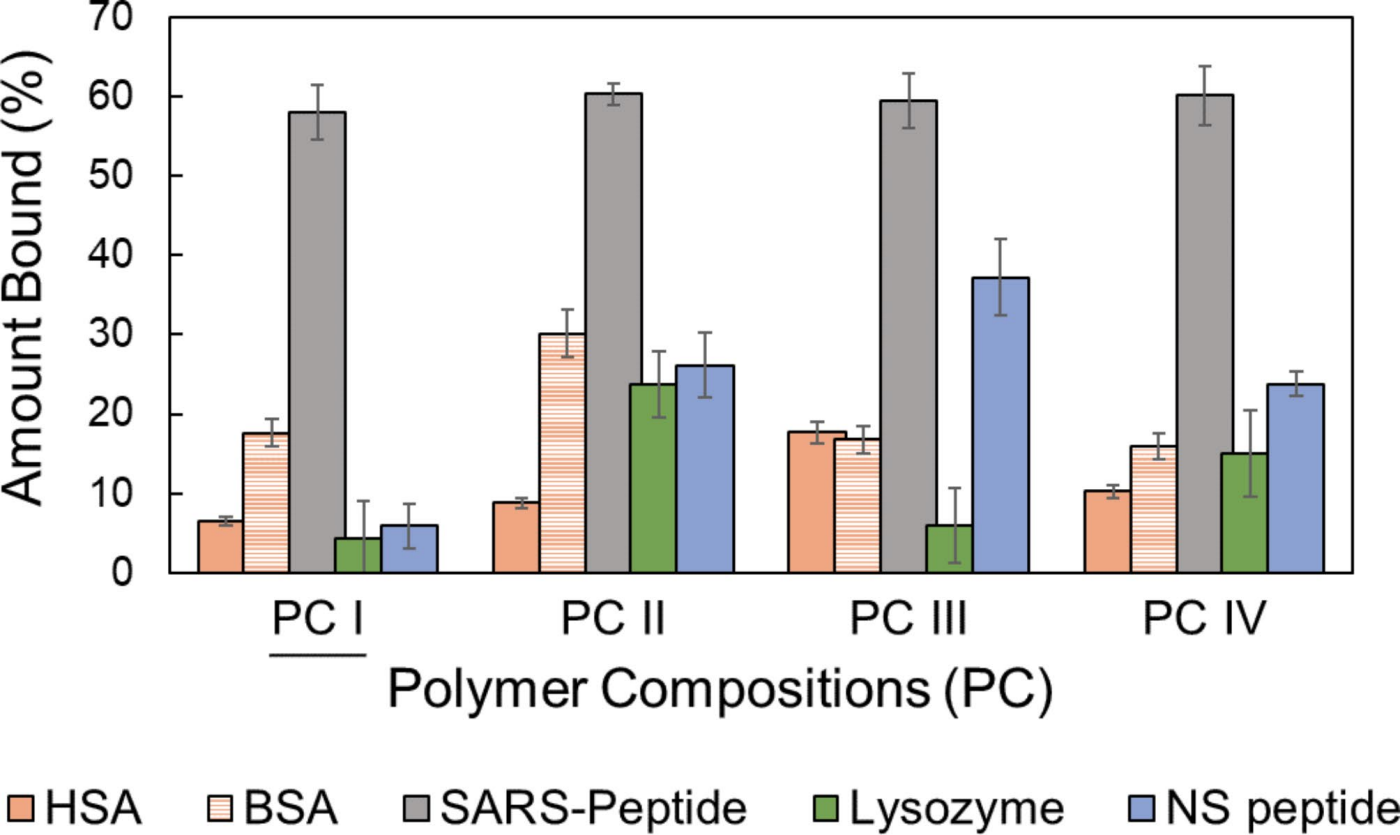
Specificity studies with human serum albumin (HSA), bovine serum albumin (BSA), lysozyme and a non-specific epitope/peptide relevant to other viruses (NS peptide) compared to SARS-CoV-2 specific peptide/epitope bound to MIPs. Clearly, PC I has the lowest amount of non-specific interactions with other proteins. The performance of rationally selected PC I can be applied for testing with the source protein (Spike) or the entire virus (SARS-CoV-2). Synthesis and experiments were performed in triplicates. Error bars indicate standard deviation (±SD).

Therefore, we examined the binding interactions of PC I-IV with other non-specific proteins to analyse how IF translate to specific MIP application (Fig. 5). We selected standard proteins like human serum albumin (HSA), bovine serum albumin (BSA), lysozyme (Lys) and a peptide/epitope (NS peptide) designed from other virus’ derived proteins. In a good correlation with IF, the non-specificity was the least for PC I, except for a modest binding with BSA. Interestingly, BSA in contrast to HSA, displayed considerable binding affinity with all four PC compositions, although it is reasonable to assume that BSA is not a common contaminant in human samples. PC I has been optimally templated specific to the SARS-CoV-2 peptide whereas PC II demonstrated the highest level of non-specific binding. This can be attributed to the presence of MPTMS that can mediate strong thiol-based interactions with cysteine containing proteins. This, indeed, can be noted as a pivotal determinant for the consideration of MPTMS in MIP development (as disulphide (covalent) interactions can readily form to lead to non-specificity). PC III and PC IV that involve UPTMS also showed reasonable non-specific binding with at least two proteins. The lower binding capacity of PC I towards non-specific proteins prompted its further application to check with specific proteins and viruses.

and a non-specific epitope/peptide relevant to other viruses (NS peptide) compared to SARS-CoV-2 specific peptide/epitope bound to MIPs. Clearly, PC I has the lowest amount of non-specific interactions with other proteins. The performance of rationally selected PC I can be applied for testing with the source protein (Spike) or the entire virus (SARS-CoV-2). Synthesis and experiments were performed in triplicates. Error bars indicate standard deviation (± SD).

### Core-shell type artificial antibodies

**Fig. 6:**
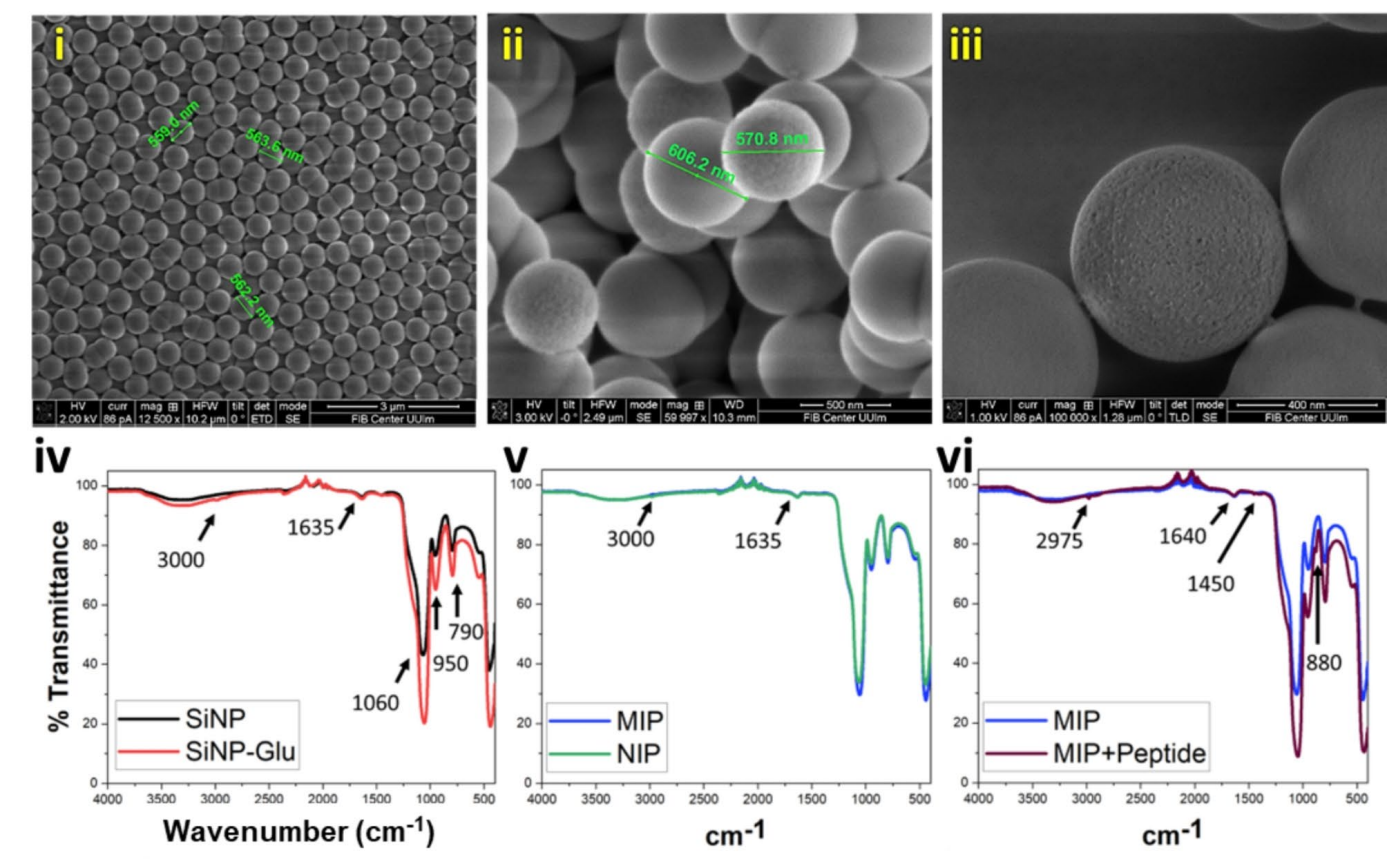
SEM characterization of (**i**) Silica Nanoparticles (SiNP), (**ii**) glutaraldehyde coated SiNP (SiNP-Glu) and (**iii**) peptide/epitope imprinted SiNP (MIP). Similarly, (**iv**) and (**v**) represent FTIR characterization after each coating step. Due to the formation of a thin polymer layer formed, the comparative differences in the spectra are less. Also, MIPs were rebound with the peptide and characterized (MIP + peptide) (**vi**). Slight differences in spectra and appearance of a new peak at 880 cm^−1^ indicate the peptide bound to the MIPs. NIP represents non-imprinted SiNP.

The imprinting was done on silica nanoparticles in order to form a ‘core-shell’ type nanocomposite system. The size of the nanoparticles was around 600 nm ± 50 nm. The principle motivation to synthesize large-sized particles was to enable binding of (more than one) viruses through their surface-expressed Spike protein. Silica nanoparticles were monodispersed and uniformly sized (Fig. 6i). After functionalization with glutaraldehyde, particles were observed with a modified surface morphology showing slightly cloudy appearance, but sharp differences could not be noted due to the thin layer of coating (Fig. 6ii). Similarly, after MIP synthesis the increased surface roughness could be observed but a highly porous surface was difficult to characterize due to the thin coat (Fig. 6iii). The peptide ideally possesses a size ranging from approximately 2–10 nm and therefore, the MIP synthesis was conducted for a short time to regulate the coating thickness, ensuring effective removal post-extraction. These surface imprinted nanoparticles enable rapid binding kinetics and increased binding efficiency and is highly advantageous over the conventional bulk imprinting process. Parallelly, NIP nanoparticles were also characterized with SEM and showed a smoother surface compared to MIP nanoparticle morphology.

Each step of functionalization was also characterized by FTIR spectroscopy to check the appearance of relevant peaks representing different chemical groups (Fig. 6iv, v and vi). Cleary, the characteristic peak of silica nanoparticles was observed at 790, 950, and 1060 cm^−1^ owing to Si-O-Si symmetric stretching, Si-OH stretching, and Si-O-Si asymmetric stretching vibrations, respectively and the surface hydroxyl groups are shown by a broad peak around 3500 cm^−1^ (Fig. 6iv). Functionalized nanoparticles obtained after glutaraldehyde coating (SiNP-Glu) showed new peaks around 3000 cm^−1^ representing symmetric/asymmetric stretching from the –CH (alkane) group. Peaks between 1500 and 1700 cm^−1^ indicate the presence of imine bonds (-C = N) and carbonyl groups (-C = O) resulting from glutaraldehyde attachment. Due to the thin layer of coating, the peaks are not very dominant and also tend to coincide because of similar functional groups yet, mark observable differences after every coating. Similarly, after MIP and NIP synthesis, differences in the intensity are clearly visible indicating presence of a subsequent coating (Fig. 6v). We also tested the binding of the peptide with the MIP nanoparticles (Fig. 6vi). First, the particles were incubated with the peptide and then, washed to remove the unbound peptide. The particles were centrifuged and dried overnight for subsequent use in FTIR characterization. Appearance of new peaks between 1300 and 1500 cm^−1^ and around 2975 and 880 cm^−1^ (relating to presence of amide linkages and aromatic amino acids) along with increase in the intensity of existing peaks indicated the peptide bound silica nanoparticles.

Next, we characterized the surface properties of the nanoparticles for their zeta potential that represent change in the charge based on appearance of different functional groups after every step. Clearly, silica nanoparticles have a negative surface charge that changed to positive after APTES functionalisation (Table 4). Post-glutaraldehyde attachment, the potential decreased to lesser positive value of around + 15.63. Clear differences were noted after MIP synthesis for every different type of PC taken into consideration. For example, compositions employing APTES as a monomer (PC I, III and VII) showed highest positive value of the zeta potential and compositions with only hydrophobic groups (PC V and VI) didn’t see a greater increase compared to glutaraldehyde coated MIPs. There were also some contrasting trends based on the monomers used, like the composition PC II including MPTMS with APTMS showed comparatively lesser surface charge (+ 9.3) as opposed to PC VII having MPTMS with APTES than APTMS. The effect of MPTMS on the zeta potential is more overshadowed by amino groups from APTES. This can be further studied to account for differential hydrolysis rate of APTMS and APTES. The surface may also differ based on how these monomers are arranged on the surface or are embedded in the cavities. Similarly, the NIP of PC I (+ 21.7) shows similarity to PC VIII (+ 18.8) in terms of lesser positive charge unlike MIP-PC I (+ 28), again representing ambiguous arrangement of charges when cavities are templated with the peptide.

Nevertheless, we currently limit our focus and conclude that different zeta potential values indicate the presence of different surface charge owing to the use of different monomer combinations. PC IV, V and VI do not show much difference in the potential compared to glutaraldehyde coated nanoparticles mostly due to neutral contribution to the surface charge from respective monomers.

**Table 4:** Zeta potential of silica nanoparticles after each modification step. MIPs formed using different polymer compositions (PC I to VIII) show clear differences in their surface charge/potential. This confirms the presence of different functionalities on the surface of each type of MIP.

Nanoparticle	Zeta potential (mV)
Silica nanoparticles (SiNP)	-48.27 ± 1.13
APTES coated SiNP (SiNP-APTES)	+ 30.2 ± 0.1
Glutaraldehyde functionalized SiNP (SiNP-Glu)	+ 15.63 ± 0.45
MIP (PC I)	+ 28.03 ± 0.87
NIP (PC I)	+ 21.67 ± 1.11
MIP (PC II)	+ 9.31 ± 0.37
MIP (PC III)	+ 29.23 ± 1.56
MIP (PC IV)	+ 18.80 ± 1.56
MIP (PC V)	+ 17.40 ± 0.22
MIP (PC VI)	+ 17.47 ± 0.46
MIP (PC VII)	+ 31.50 ± 1.34
MIP (PC VIII)	+ 18.83 ± 1.35

We also characterized the nanoparticles with EDX to confirm the elemental composition. Figure 2S, SI shows ~ 64% Si and ~ 34% O representing the nature for the SiO_2_ nanoparticles. As the peaks of Si and O contributed to the major percentage of the elements and the MIP/NIP layer was very thin to mark substantial differences in C and N content therefore, we did not use EDX analysis further for the remaining functionalized nanoparticles.

### Artificial antibody binding with virus-like particles

In a further proof-of-concept study, we used virus-like-particles (VLP) containing all structural proteins of SARS-CoV-2 including Spike (Fig. 5S, SI) to check their binding with MIP/NIP nanoparticles (PC I)^60,61^. For this, MIP nanoparticles were mixed and allowed to bind VLP followed by centrifugation that left two fractions: first, with nanoparticles bound to VLP in the pellet and second, unbound VLP in the supernatant. We used fluorescamine dye with both fractions and visualised the first fraction using fluorescence microscopy. For quantitative assessment of the binding events, the second fraction was employed for spectrophotometric analysis. Figure 3Sa) demonstrates the binding ability of MIP for VLP and Fig. 3S b) shows only MIP nanoparticles incubated with fluorescamine. Due to the ability of this dye to react with primary amines, nanoparticles having APTES/APTMS-based amino groups (PC 1) exhibited minimal fluorescence signal. However, there was a clear difference in florescence intensity and particle assembly to mark the binding events with VLP. Parallely, we performed a quantitative evaluation using spectrophotometry which involved comparing the supernatants derived from MIPs and NIPs for discerning binding events (Fig. 4S, SI). Clearly, a substantial difference in binding was observed for MIPs in comparison to NIPs, thus, corroborating the efficacy of peptide imprinting strategy. This substantiates that the specificity of peptide-templated MIPs is indeed adequate for capturing intact protein containing viruses or virus-like-particles.

### Discussion and conclusions

Molecularly imprinted polymers (MIPs) present a promising alternative to conventional immunoassays, overcoming reliance on antibodies and similar detection methods. Customizable for specific target molecules or biomarkers, MIPs benefit from enhanced design precision through computational interventions. However, achieving specificity comparable to natural antibodies necessitates focused efforts, particularly in refining *in silico* design approaches. This strategically mitigates the need for time-consuming trial-and-error methods, expediting advancements towards the next generation of precision MIPs.

In this study, we introduce SARS-CoV-2-selective MIPs as a model to pioneer a novel computational design strategy. Deriving from the advancements in epitope imprinting that eliminates the risk of handling the entire – potentially infectious – virus or the viral proteins, we have selected an epitope based on the Spike protein of the virus. This model showcases versatility, offering a time-efficient approach and demonstrating applicability for developing artificial antibodies against various epitopes or mutations. This is especially beneficial in dealing with rapidly mutating viruses, where healthcare technologies must swiftly adapt and update accordingly. MMSD offers a streamlined pathway for developing diagnostic and therapeutic tools against evolving SARS-CoV-2 variants. Epitope-specific MIPs exhibit binding capabilities to the template, both independently and as part of the source protein or the virus itself, as evidenced in our proof-of-concept study involving virus-like particles. By imprinting a smaller template (i.e., epitope) compared to the entire protein or biological species, MIPs with imprinted binding sites enable higher precision and reduced cross-reactivity.

The novelty of this study lies in the combinatorial screening technique that utilises multiple monomers to selectively template the MIPs. The focus is to closely mimic the specificity structuring observed in antibodies. Based on the diverse array of amino acids that contribute to non-covalent interactions at the antibody-antigen interface, a strategic intervention at the initial phase of MIP design is imperative—specifically in the selection of monomers. We have meticulously modeled a sophisticated multi-monomer-based library for MIP design, especially suited for biomolecular templates. This approach represents an improvement over the conventional practise of conducting individual monomer screenings and subsequently employing arbitrary combinations of monomers for subsequent synthesis.

An ideal monomer combination should facilitate a broad spectrum of non-covalent interactions, encompassing H-bonding, hydrophobic, and van der Waals interactions. Hence, combinatorial screening is crucial to understand their combined effects on the template and on each other. The MMSD approach allows for the identification of cooperative effects over antagonistic monomer interactions, ultimately guiding experimental synthesis. This approach has the potential to revolutionize monomer selection, enabling the screening of a larger number of combinations within a reduced timeframe. The MMSD method establishes an advanced assessment of the pre-polymerization complex that directly reflects on the MIP performance. This outcome stems from the hierarchical arrangement of predicted binding affinities for the 36 systematically designed combinations and their corresponding proportional impact on the binding capacity and imprinting factor of the synthesised polymers. *In silico* guided organic silanes-based polymer compositions were combined with chemically compatible silica nanoparticles in a surface imprinting approach. Simultaneously we could innovate a fluorescent assay format based on fluorescamine to introduce high throughput and rapid binding analysis of MIPs in 96 well plates.

The notweworthy findings in this study could help establish the correlation in binding energy scores and simulated interactions with the binding capacity and imprinting factor of the polymer. Some monomers may establish strong affinty with the entire template surface, leading to high binding capacity but also generating non-specificity. To address this, distinct functional monomers should be strategically arranged or distributed in proximity to each other within the polymer structure, allowing for a comprehensive and complementary interaction with the targeted template. This diversity in the chemical composition facilitated by the novel MMSD method allowed to improve imprinting factors, emphasizing its impact on specificity.

MMSD can also be used to explore the dynamics of polymerization mixtures. For instance, methoxysilanes can hydrolyze to silanols at different rates than ethoxysilanes, thus necessitating the characterization of sol-gel chemistry. In systems with multiple monomers, each exhibiting distinct hydrolysis kinetics, MMSD can effectively simulate these complex interactions. By initially focusing on simulating monomers in their pre-hydrolyzed state, we optimized computational efficiency and validated the method using a smaller dataset. These early, simplified simulations provide a robust foundation for progressing to polymer-level modeling, where hydrolyzed species and their roles in the polymer matrix can be more comprehensively analyzed.

In conclusion, MMSD creates an infinite number of opportunities to simulate MIPs via suitable approximation of monomer effects leading to MIPs with indeed antibody-like binding properties. Additional case studies will explore screening methods involving combinations of three, four, five or more monomers with or without consideration of underlying interaction assumptions yielding data sets that may then be further processed via machine learning (ML) techniques for limiting the number of confirmatory experimental studies.The scope of the research may be expanded to include other emergent diseases along with specific epitopes, templates, and proteins. Furthermore, computational methodologies such as molecular dynamics (MD) simulations may provide supplementary validation of the findings derived from MMSD predictions. MD simulations can be integrated to assess the stoichiometric ratios of the best monomer combinations identified through MMSD screening. Therefore, MMSD stands out as the foundational step guiding the entire computational and experimental design of MIPs. Additionally, the monomer library can be innovated to select combinations of electroactive monomers, paving the way for the development of MIP-based sensors. Such sensors have seen increased application in enhancing system sensitivity. Similarly, the selection of biocompatible monomers can enable the *in vivo *applicability of MIPs.

## Electronic supplementary material

Below is the link to the electronic supplementary material.

Supplementary Material 1

## Data Availability

The datasets used and/or analysed during the current study available from the corresponding author on reasonable request.
