# Supplementary material for: Rational design based on multi-monomer simultaneous docking for epitope imprinting of SARS-CoV-2 spike protein
Source: Sci Rep. 2024 Oct 4;14:23057. doi: 10.1038/s41598-024-73114-3 (PMC11452659; doi:10.1038/s41598-024-73114-3)
Supplement: Supplementary file 1 — Supplementary Material 1 [file 41598_2024_73114_MOESM1_ESM.docx]

Supplementary Information

Rational design based on multi-monomer simultaneous docking for epitope imprinting of SARS-CoV-2 Spike protein

Soumya Rajpal^a,c^, Alex D. Batista^a,d^, Rüdiger Groß^b^, Jan Münch^b^, Boris Mizaikoff^a,d *^, Prashant Mishra^c *^

*^a^ Institute of Analytical and Bioanalytical Chemistry (IABC), Ulm University, Albert-Einstein-Allee 11, 89081 Ulm, Germany*

*^b^ Institute of Molecular Virology (IMV), Ulm University Medical Center, Meyerhofstraße 1, 89081 Ulm, Germany*

*^c^ Department of Biochemical Engineering and Biotechnology, Indian Institute of Technology Delhi, New Delhi 110016, India
^d^ Hahn-Schickard, Sedanstraße 14, 89077 Ulm, Germany*

*Corresponding authors: [boris.mizaikoff@uni-ulm.de](mailto:boris.mizaikoff@uni-ulm.de), [pmishra@dbeb.iitd.ac.in](mailto:pmishra@dbeb.iitd.ac.in)

**Table of Contents Page No.**

MD simulations of the peptide structure...................................................................................................S-1

Multi-monomer simultaneous docking calculations…………………………….……………..………. S-2

Monomer-Peptide Interaction mapping……………………………………………….……………..… S-3 Particle Characterization ………………………………………………………...…………………….. S-5

Binding with virus-like particles………………………………………………………...…………… ...S-6

Materials and Methods ………………………………………………………..………...…………… ...S-7

Preparation of SARS-CoV-2 virus-like-particles (VLPs)………………………………………………S-9

References ………………………………………………………...………………………………… ...S-11

**
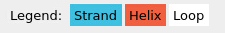
**

**
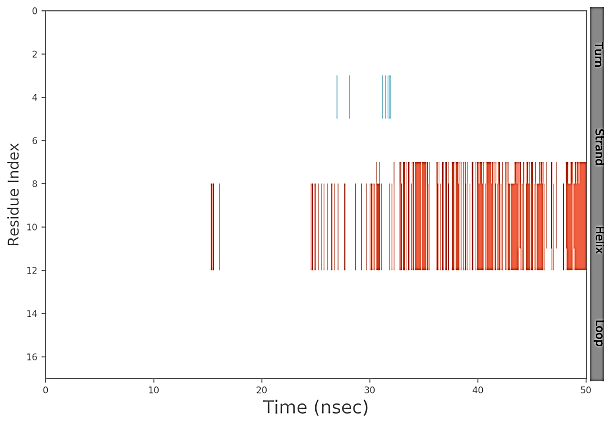
**

Figure 1S: Peptide secondary structure elements (SSE) like alpha-helices and beta-strands are monitored throughout the MD simulation over 50 nanoseconds in a simulation box of water with a salt concentration of 0.1M. The plot above monitors each residue and its SSE assignment over time. The peptide is majorly an extended random coil like structure with few residues forming helix like conformation.

**Multi-monomer simultaneous docking (MMSD) calculations**

Table 1S: Multi-monomer simultaneous docking (MMSD) scores for 36 monomer combinations used and compared with SMD.

Every monomer’s docking score (mean binding energy of the first cluster) was extracted from the respective clustering histogram in the .dlg files generated by Autodock 4. To compare the changes in the binding affinity of the combination from the individual monomer binding, a cumulative of the MMSD scores was tabulated and to create a similar SMD cumulative, respective single monomer scores were used from Table 1. The difference of the SMD and MMSD sums were used as an indicator to observe the combinations that have seen the significant changes (negative or positive). For example, the monomer combination 2 has seen a negative effect when applied in simultaneous docking whereas combination 34 has seen the maximum increase indicating that the monomer(s) comprised in the combination have improved binding effect.

|  | Monomer/Polymer Combination | Respective monomer binding energy (∆G =-kcal/mol) | | | | Sum of MMSD scores for respective combination | Sum derived from SMD scores | Difference |
| --- | --- | --- | --- | --- | --- | --- | --- | --- |
| 1 | PTES, DIDMS, MTMS, TEOS | -3.32 (PTES) | -2.77 (DIDMS) | -2.42 (MTMS) | -2.18 (TEOS) | = -10.69 | -11.34 | -0.65 |
| 2 | PTES, DIDMS, IBTES, TEOS | -3.30 | -2.75 | -2.41 | -2.19 | -10.65 | -11.36 | -0.71 |
| 3 | PTES, DIDMS, UPTMS, TEOS | -3.33 | -2.74 | -2.76 | -2.35 | -11.18 | -11.07 | 0.11 |
| 4 | PTES, DIDMS, TMOS, TEOS | -3.32 | -2.74 | -2.22 | -2.21 | -10.49 | -10.94 | -0.45 |
| 5 | PTES, DIDMS, CETES, TEOS | -3.30 | -2.74 | -2.37 | -2.39 | -10.80 | -11.23 | -0.43 |
| 6 | PTES, DIDMS, APTMS, TEOS | -3.31 | -2.74 | -3.11 | -2.25 | -11.41 | -10.63 | 0.78 |
| 7 | PTES, DIDMS, APTES, TEOS | -3.32 | -2.77 | -2.89 | -2.25 | -11.23 | -10.74 | 0.49 |
| 8 | PTES, DIDMS, MPTMS, TEOS | -3.31 | -2.75 | -2.57 | -2.37 | -11.00 | -10.64 | 0.36 |
| 9 | PTES, MTMS, IBTES, TEOS | -3.31 | -2.49 | -2.45 | -2.22 | -10.47 | -11.09 | -0.62 |
| 10 | PTES, MTMS, UPTMS, TEOS | -3.33 | -2.51 | -2.86 | -2.35 | -11.05 | -10.80 | 0.25 |
| 11 | PTES, MTMS, TMOS, TEOS | -3.31 | -2.51 | -2.22 | -2.23 | -10.27 | -10.67 | -0.40 |
| 12 | PTES, MTMS, CETES, TEOS | -3.32 | -2.51 | -2.44 | -2.40 | -10.67 | -10.96 | -0.29 |
| 13 | PTES, MTMS, APTMS, TEOS | -3.32 | -2.51 | -3.11 | -2.27 | -11.21 | -10.36 | 0.85 |
| 14 | PTES, MTMS, APTES, TEOS | -3.34 | -2.51 | -2.99 | -2.24 | -11.08 | -10.46 | 0.62 |
| 15 | PTES, MTMS, MPTMS, TEOS | -3.31 | -2.50 | -2.59 | -2.37 | -10.77 | -10.36 | 0.41 |
| 16 | PTES, IBTES, UPTMS, TEOS | -3.31 | -2.53 | -2.78 | -2.38 | -11.00 | -10.82 | 0.18 |
| 17 | PTES, IBTES, TMOS, TEOS | -3.33 | -2.54 | -2.31 | -2.25 | -10.43 | -10.69 | -0.26 |
| 18 | PTES, IBTES, CETES, TEOS | -3.34 | -2.57 | -2.43 | -2.39 | -10.73 | -10.98 | -0.25 |
| 19 | PTES, IBTES, APTMS, TEOS | -3.30 | -2.54 | -3.12 | -2.24 | -11.20 | -10.38 | 0.82 |
| 20 | PTES, IBTES, APTES, TEOS | -3.31 | -2.58 | -2.94 | -2.24 | -11.07 | -10.49 | 0.58 |
| 21 | PTES, IBTES, MPTMS, TEOS | -3.32 | -2.55 | -2.56 | -2.36 | -10.79 | -10.39 | 0.40 |
| 22 | PTES, UPTMS, TMOS, TEOS | -3.32 | -2.88 | -2.26 | -2.14 | -10.60 | -10.40 | 0.20 |
| 23 | PTES, UPTMS, CETES, TEOS | -3.33 | -2.76 | -2.41 | -2.17 | -10.67 | -10.69 | -0.02 |
| 24 | PTES, UPTMS, APTMS, TEOS | -3.33 | -2.78 | -3.07 | -2.16 | -11.34 | -10.09 | 1.25 |
| 25 | PTES, UPTMS, APTES, TEOS | -3.31 | -2.76 | -2.87 | -1.96 | -10.90 | -10.20 | 0.70 |
| 26 | PTES, UPTMS, MPTMS, TEOS | -3.31 | -2.74 | -2.55 | -2.14 | -10.74 | -10.10 | 0.64 |
| 27 | PTES, TMOS, CETES, TEOS | -3.31 | -2.21 | -2.38 | -2.39 | -10.29 | -10.56 | -0.27 |
| 28 | PTES, TMOS, APTMS, TEOS | -3.32 | -2.21 | -3.11 | -2.25 | -10.89 | -9.96 | 0.93 |
| 29 | PTES, TMOS, APTES, TEOS | -3.30 | -2.23 | -2.99 | -2.26 | -10.78 | -10.07 | 0.71 |
| 30 | PTES, TMOS, MPTMS, TEOS | -3.32 | -2.22 | -2.59 | -2.26 | -10.39 | -9.97 | 0.42 |
| 31 | PTES, CETES, APTMS, TEOS | -3.32 | -2.40 | -3.15 | -2.37 | -11.24 | -10.25 | 0.99 |
| 32 | PTES, CETES, APTES, TEOS | -3.32 | -2.35 | -3.00 | -2.37 | -11.04 | -10.36 | 0.68 |
| 33 | PTES, CETES, MPTMS, TEOS | -3.31 | -2.35 | -2.48 | -2.22 | -10.36 | -10.26 | 0.10 |
| 34 | PTES, APTMS, APTES, TEOS | -3.32 | -3.05 | -2.95 | -2.29 | -11.61 | -9.76 | 1.85 |
| 35 | PTES, APTMS, MPTMS, TEOS | -3.32 | -3.05 | -2.56 | -2.30 | -11.23 | -9.66 | 1.57 |
| 36 | PTES, APTES, MPTMS, TEOS | -3.32 | -2.94 | -2.54 | -2.28 | -11.08 | -9.76 | 1.32 |

**Monomer-Peptide Interaction mapping**

Table 2S: 2D interaction map representing some common set of interactions with the peptide at multiple residues derived from MMSD calculations.

Green dotted lines represent hydrogen bonding, dark pink represents π–π bonds interactions, light pink lines show π–alkyl and alkyl-based interactions, and purple lines indicate π–sigma interactions. Blue areas represent solvent-accessible surfaces. Light green highlighted residues (existing independently) mark van der Waals interactions. Atoms are labelled for oxygen (O, red), silica (Si, grey), nitrogen (N, blue), sulphur (S, yellow) and hydrogen (H, black).

| 1. PTES | |
| --- | --- |
| 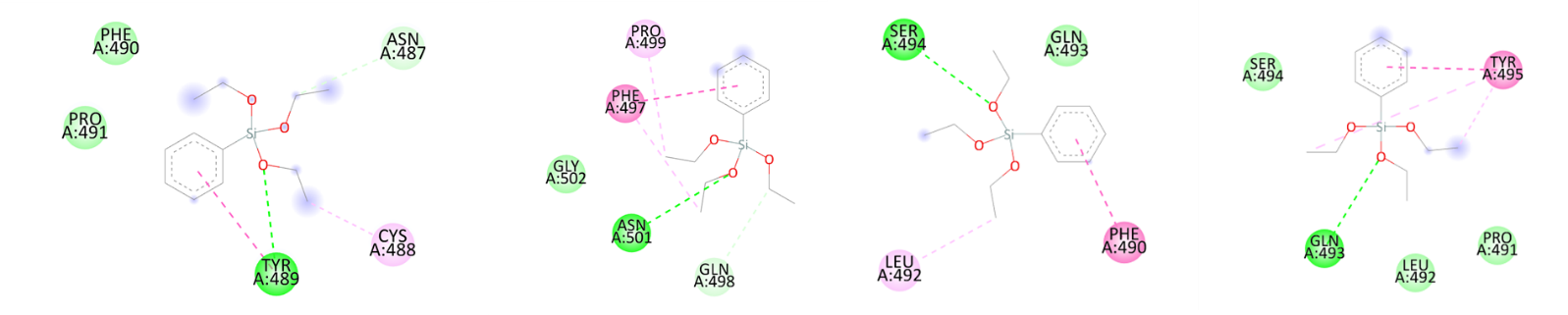  **i ii iii iv** | |
| 1. DIDMS | |
| 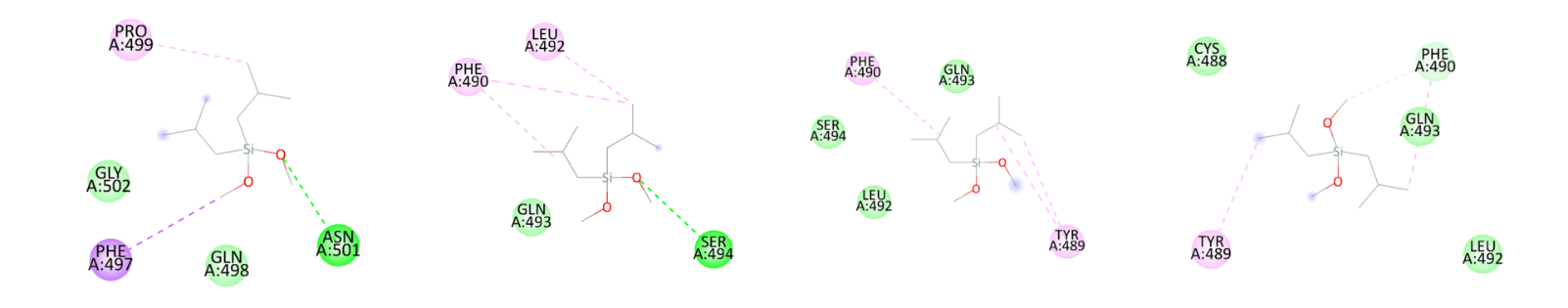  **i ii iii iv** | |
| 1. APTMS | |
| 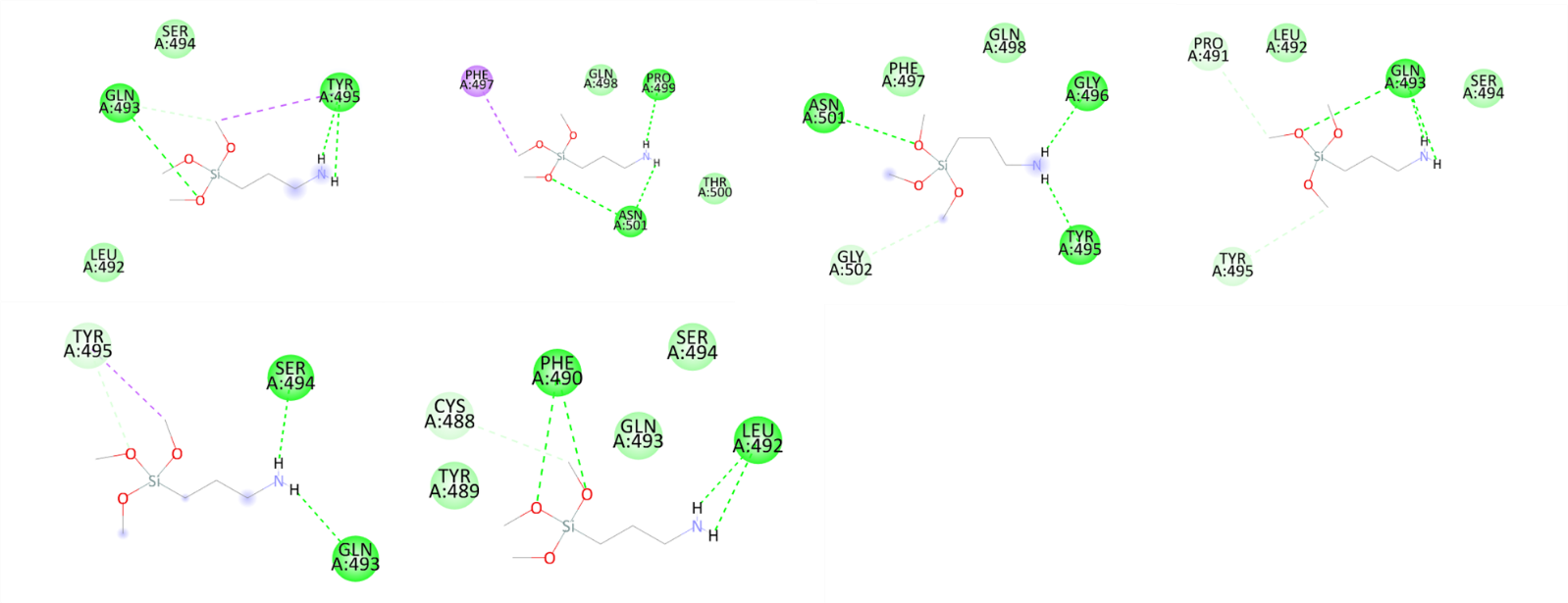  **i ii iii iv**  **v vi** | |
| 1. APTES | |
| 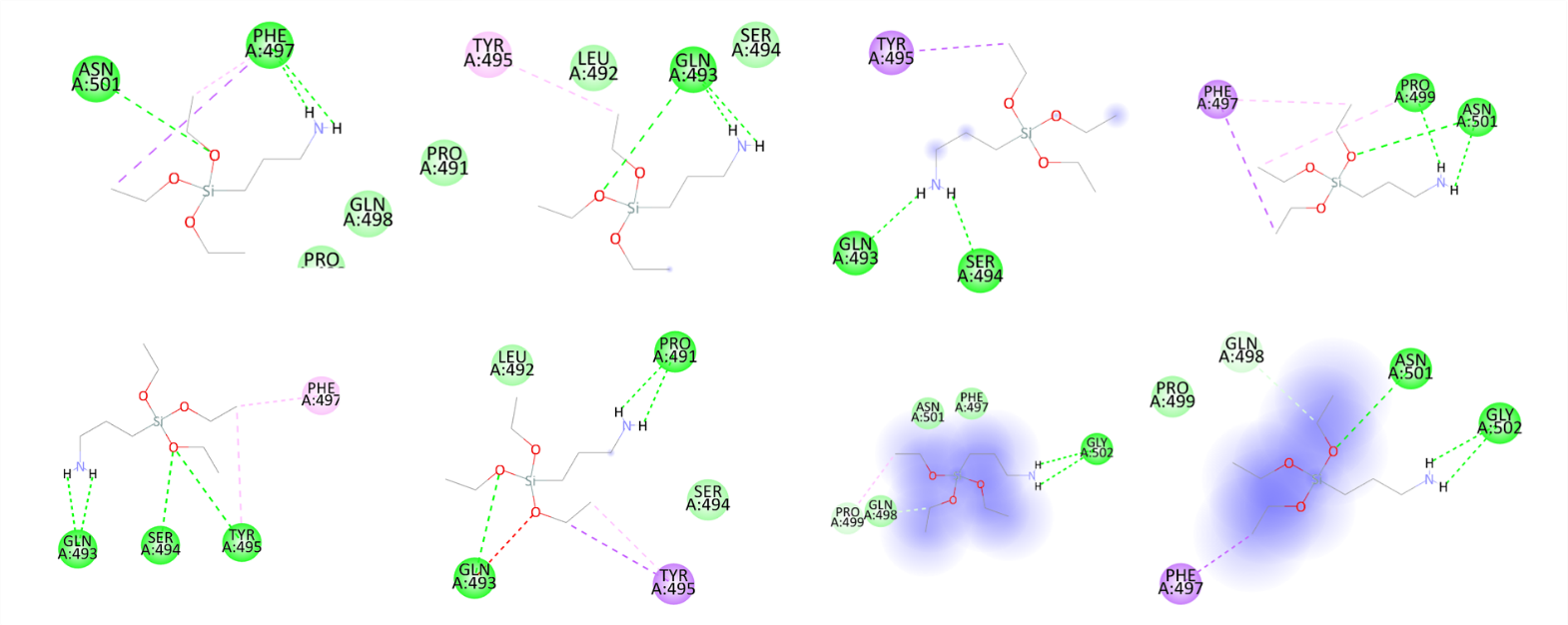  **i ii iii iv**  **v vi vii viii** | |
| 1. UPTMS | |
| 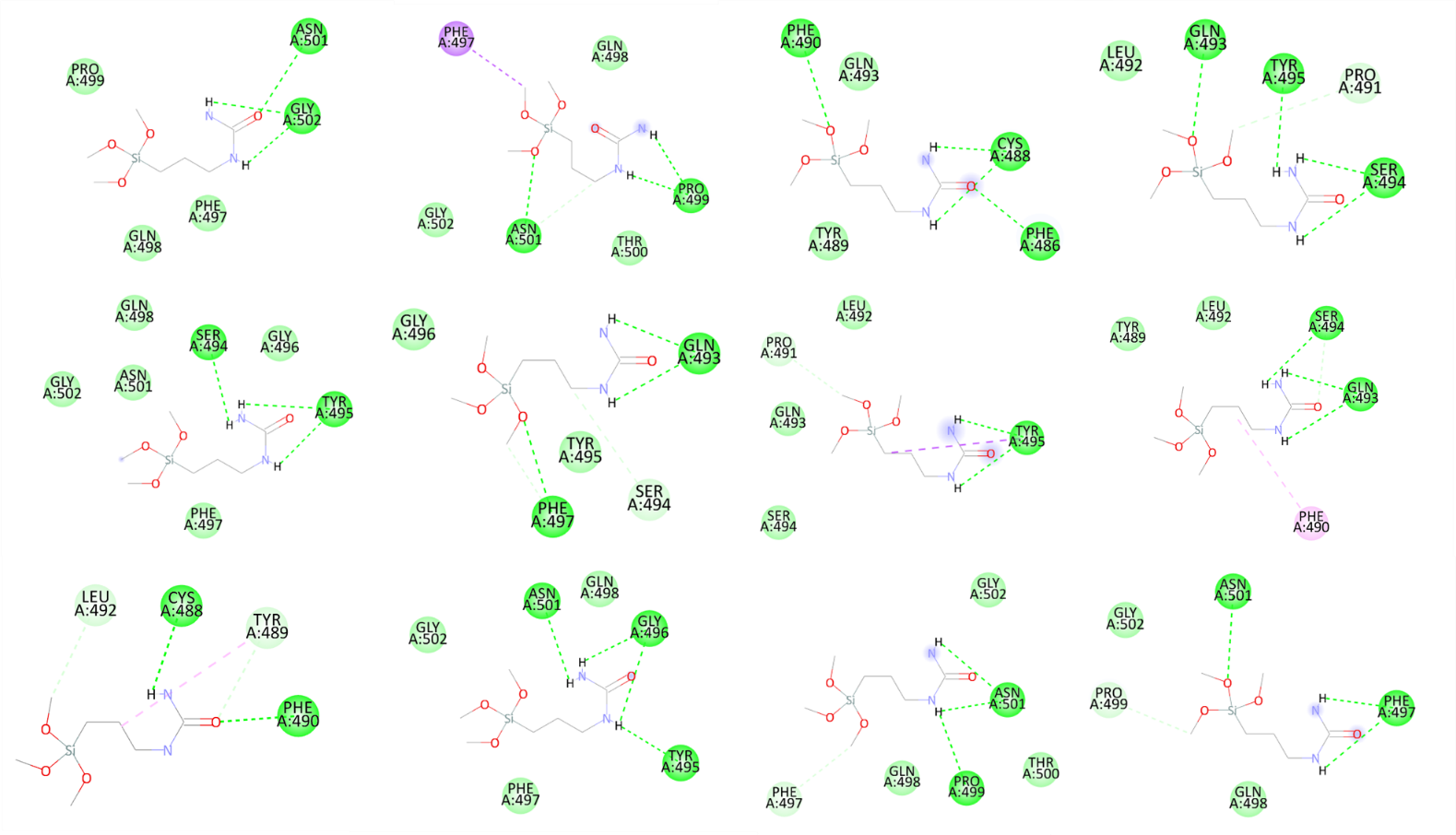 | |
| 1. MPTMS | |
| 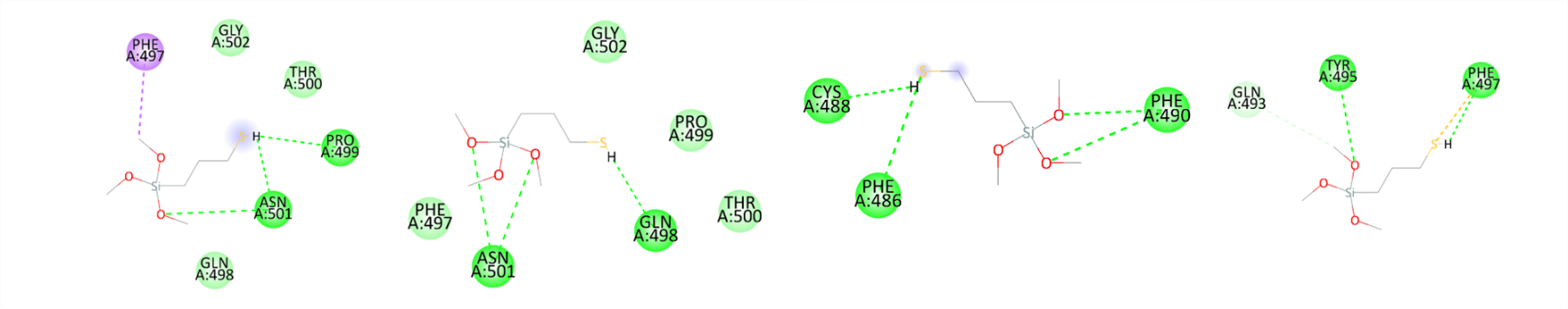  **i ii iii iv** | |
| 1. MTMS | 1. IBTES |
| 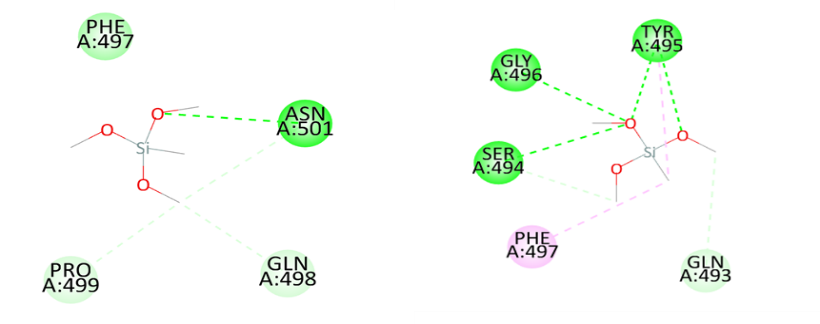  **i ii i** | 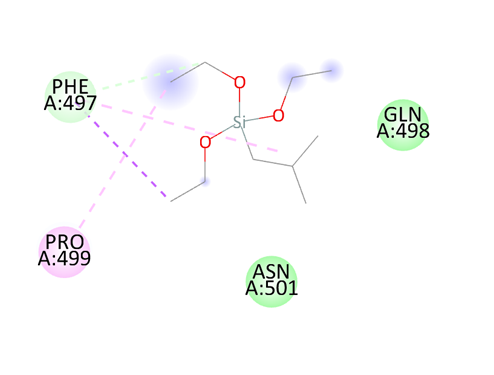 |

**i ii iii iv**

**v vi vii viii**

**ix x xi xii**

**Particle characterization with EDX**


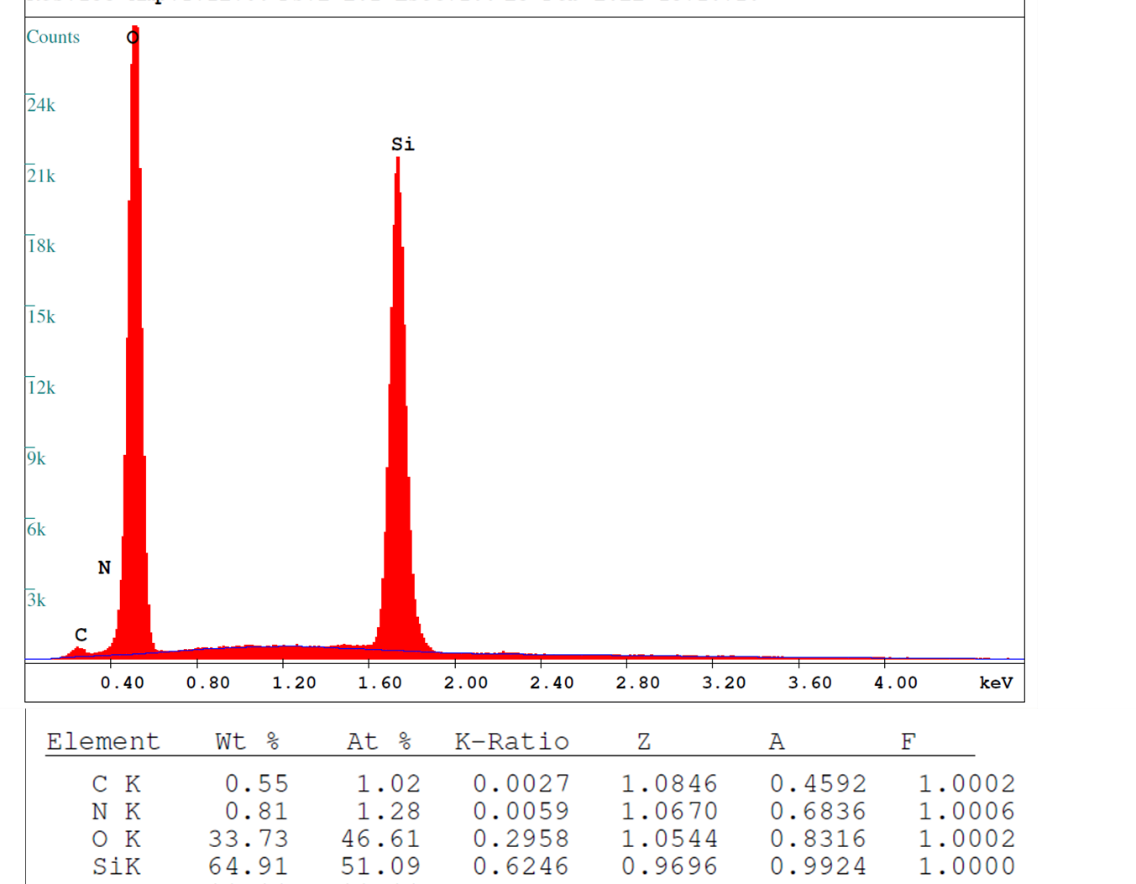


Figure 2S: EDX of SiNPs confirming the presence of Si and O characteristic of silicon dioxide

**Binding with virus like particles**


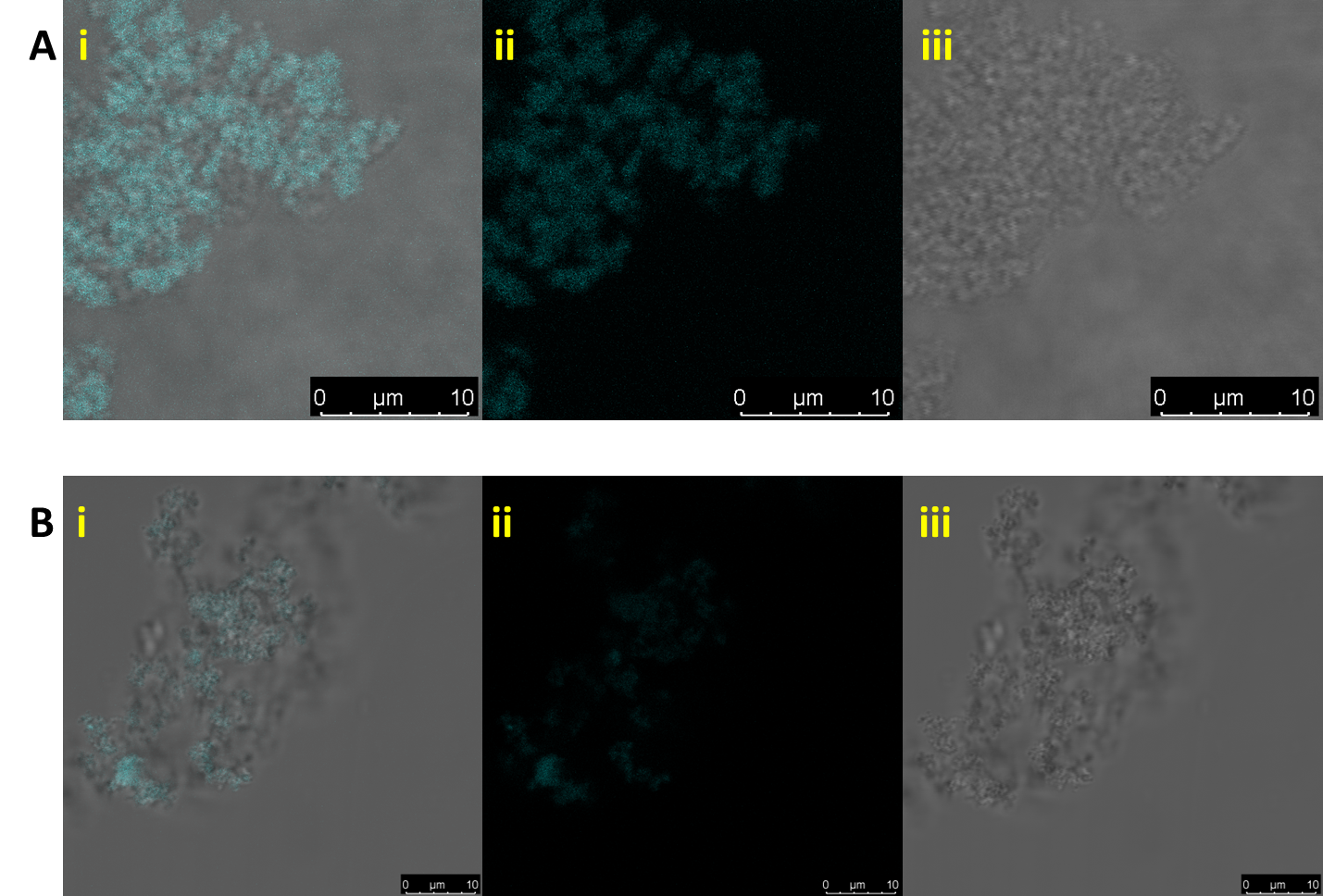


Figure 3S**:** A: MIPs bound with virus like particles (VLPs). B: MIPs without VLPs (i) Overlay of (ii) fluoresence and (iii) bright field images). Fluorescent labelling with fluorescamine dye. Minimal fluorescence is obtained with particles itself due to occurrence of amino groups from APTES monomer employed during synthesis.

**

Figure 4S: Analysis of binding of MIPs and NIPs of PC 1 with virus like particles based on fluorescence intensities. Experiment done in triplicates. Concentration of Initial VLP solution ~10^10^ particles/mL. (p<0.01). Error bars indicate standard deviation (±SD).

**Materials and Methods**

**Materials**

PTES, APTES, APTMS were purchased from Alfa Aesar (Germany). UPTMS, IBTES was purchased from abcr Chemie (Germany). DIDMS, ammonium hydroxide solution (28.0–30.0% NH_3_ basis), PBS buffer (pH 7.4), and glutaraldehyde were purchased from Sigma Aldrich (Steinheim, Germany). MPTMS, TEOS was obtained from Merck (Darmstadt, Germany). The custom peptides FNCYFPLQSYGFQPTNG and KLPDDFTGCV were obtained from GenScript (Leiden, The Netherlands). Fluorescamine was purchased from Merck. 96 well plates (Black) were purchased from Greiner Bio-One (Kremsmünster, Austria). Proteins including albumin from human serum, lysozyme from chicken egg white were purchased from Sigma Aldrich, USA

**Equipment**

The morphology of the particles and their composition were investigated using a Quanta 3D FEG SEM equipped with a focused gallium ion beam (FIB) (FEI Corp., Eindhoven, The Netherlands), and an EDX detector (Apollo XV SDD, EDAX). Zeta potential measurements were performed with a Zetasizer NANO ZSP (Malvern, Herrenberg, Germany). Fluorescence spectroscopy measurements were conducted using Tecan Infinite M1000 PRO plate reader (Mannedorf, Zurich, Switzerland). Fluorescence microscopy was done using Leica DMi8 system.

**Computational Simulations**

The structural files for all silane-based monomers were obtained from PubChem databank and prepared using Autodock. The epitope/peptide structure (FNCYFPLQSYGFQPTNG) was extracted from the crystal structure of the SARS-CoV-2 receptor-binding domain (ID:7JMO) using UCSF Chimera [1]. Autodock tools were used for molecular docking. Autodock is a commonly used open-source molecular docking software based on the AMBER force field suitable for proteins, nucleic acids, and other organic molecules [2].

The ligand files were converted to pdbqt files after setting the torsional degrees of freedom based on the detected rotatable bonds. For docking of silane molecules, the parameters for Si were added in the AD4.1_bound and AD4_parameters data files. Polar hydrogens were added to the peptide. Any water molecules were removed. A grid box of the dimensions 62 × 98 × 66 Å was centered around the peptide. The number of energy evaluations was set to the maximum (25 million evals) to improve the reproducibility and accuracy of the calculations. Furthermore, the number of docking runs was set to 100, specified by the ga_run parameter. For molecular docking, the Lamarckian Genetic Algorithm (LGA) was used. Docking results were analyzed using BIOVA Discovery Studio Visualizer software and UCSF Chimera [3].

Multi monomer simultaneous docking: For MMSD calculations, the scoring functions of Autodock 4 were employed. Firstly, a hierarchical sorting of the single monomer docking results based on the total binding energy (ΔG, kcal/mol) was done. Then, a set of MMSD combinations were generated keeping PTES and TEOS common in each case (Figure 2). Each combination consists of four monomers to introduce optimum number of functionalities for interaction with the target peptide. The docking parameter files (.dpf) of the monomers according to the MMSD combinations mentioned in Table 1S, SI were merged together, and docking was performed with the standard LGA method.

MMSD allows multiple conformations of every four-monomer sets to interact with the peptide at the same time. Each monomer is randomly initialized with its own set of variables [4]. The docked models were visualized using UCSF chimera.

**Silica Particle Synthesis and Functionalization**

Silica particles were synthesized based on the Stöber method. This was followed by amino functionalization using APTES. Post-treatment, the exposed NH_2_ groups formed due to APTES are subsequently used to attach glutaraldehyde on the surface of SiNPs. Glutaraldehyde functionalized SiNPs enable the easy immobilization of the peptide through its N-terminal using imine chemistry. The procedures were referred from [5]

**Synthesis of MIP and NIP**

Before MIP synthesis, the peptide is immobilized on the surface of glutaraldehyde coated SiNPs. For this, 50 mg particles are resuspended in PBS buffer (5.0 mL, pH 7.4) using mild sonication and peptide solution (250 µL, 1 mg mL^−1^) was added into the suspension and incubated for 1 h in an incubator-shaker set at 700 rpm, 10 ºC stirring. Then, the monomers in the polymer combinations 1,2,3,4,5,6,7 and 8 were added in equal molar ratios (40 moles) in the same order and incubated for 80 minutes with the temperature increased to 20 ºC. NIPs were also prepared similarly, however the peptide was replaced with PBS buffer.

Particles were centrifuged at 5500 rpm for 15 minutes followed by washing with HCl solution (15 mL, 0.1 N) using mild sonication and incubation for 10 minutes on a shaking platform. Particles were separated with centrifugation followed by a wash with AA solution (Acetonitrile: Acetic acid (9:1) and then with MilliQ water (5-7 times) to remove any residual acid. Synthesis of MIP and NIP were repeated three times for each set to reliably check for their binding performance.

**Binding Studies**

The binding studies were performed as follows: MIP or NIP (10 mg) were suspended into PBS buffer (900 µL, pH 7.4) and kept under stirring for 10 min, followed by the addition of the peptide solution (100 µL, 1 mg mL^−1^). Binding studies were done in triplicates. The mixture was kept under constant stirring in a rocking platform for 1 h at room temperature. The particles were separated by centrifuging at 5500 rpm for 15 min. The supernatant was analysed using the fluorescamine assay. For this, 200 µL was collected (also, in triplicates) from each supernatant and added in 96 well plates. Just before the measurements, 50 µL fluorescamine dye (5mg/mL, DMSO) was added in each well and the reading were taken in Tecan Infinite M1000 PRO plate reader. The plate was shaken for ~1 minute and incubated in the dark for 10 minutes. The measurement was executed upon excitation at λ_ex_ = 400 nm and emission was captured at λ_em_ = 460 nm. Multiple reads per well were recorded and averaged. A standard graph in the range of 20-100 µg/ mL for the SARS peptide was obtained and corresponding to this, the gain value was set to 57 for the SARS peptide and was set to ‘optimal’ when other peptides and proteins were analyzed. Fluorescamine is commonly used to assay the amount of peptides and proteins in the sample and works on the principle of reaction with primary amines. As shown in Figure 5S, free fluorescamine is non-fluorescent and upon reaction with primary amines (present on N-terminal or lysine (K)) it produces a fluorescent product [6] .


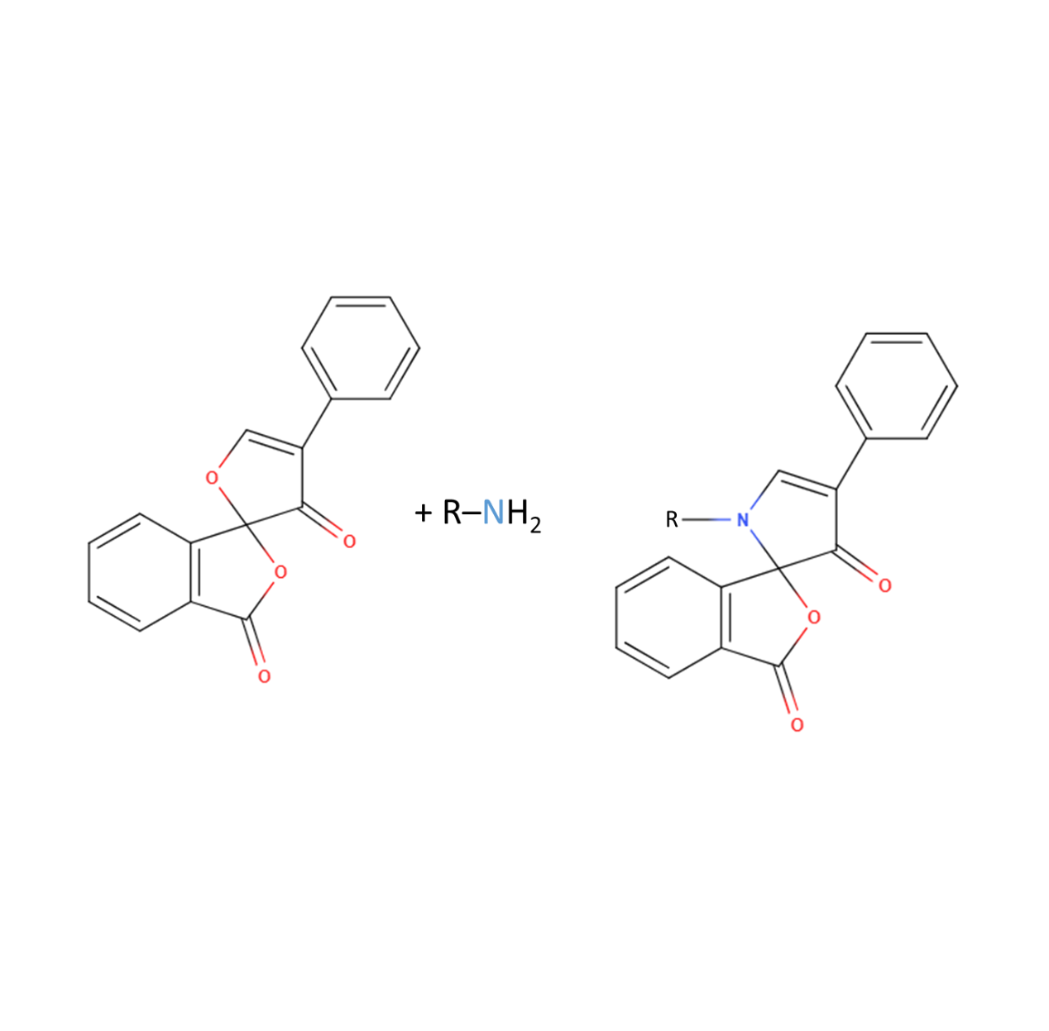


Figure 5S: Reaction of fluorescamine with a primary amine. While free fluorescamine does not show fluorescence, the product can be excited at 400 nm and emits at 460 nm. Structures were drawn using molview.org

**Preparation of SARS-CoV-2 virus-like-particles (VLPs)**

SARS-CoV-2 virus-like-particles were prepared by transient transfection of plasmids encoding all viral structural proteins as previously described with some modifications outlined below [7]. HEK293T cells were transfected with plasmids encoding SARS-CoV-2 Spike (S), membrane (M), envelope (E), and nucleocapsid (N) protein at a molar ratio of 8/6/8/3 using Transit LT-1 (Mirus) [8]. HEK293T were cultured in Dulbecco’s modified Eagle’s medium (DMEM) which was supplemented with 10% heat-inactivated foetal calf serum (FCS), 100 units/ml penicillin, 100 µg/ml streptomycin, 2 mM L-glutamine. Following transfection, cells were incubated for 2 days before the supernatant was harvested, cleared by centrifugation (1000 x g/10 min/4 deg C) then again 2000 x g/10 min/4 deg C) and filtered through a 0.45 μΜ pore size vacuum filter. The VLP-containing medium was then layer onto 20% sucrose in PBS and ultracentrifuged at 140,000 x g for 2h. The supernatant was decanted and pellets resuspended in PBS, which was then layered onto a OptiPrep gradient from 54% to 10%, and centrifugation was carried out at 38,000 rpm overnight (12-14h). Fractions were then collected and diluted 1:4 in PBS before being centrifuged at 21,000 x g/3h/4 deg C. Supernatants were poured off and the pellet resuspended in PBS. For determination of the VLP-containing fraction, an aliquot of each fraction was taken for analysis by Western Blot. Samples were separated in SDS-PAGE (4-12% BisTris, Novex), semi-dry transferred to PVDF and membranes blocked with Casein Blocker as previously described [9]. V5 (#ab15828, Abcam, detecting tagged M/E/N) and SARS-CoV-2 (GTX632604, Genetex) were then detected by fluorescent secondary antibodies (StarBright, Bio). Fractions containing bands matching all structural proteins were pooled and used as purified VLPs. VLP concentration was quantified by NTA using a ZetaView TWIN (ParticleMetrix) as previously described [7] (Figure 6S).


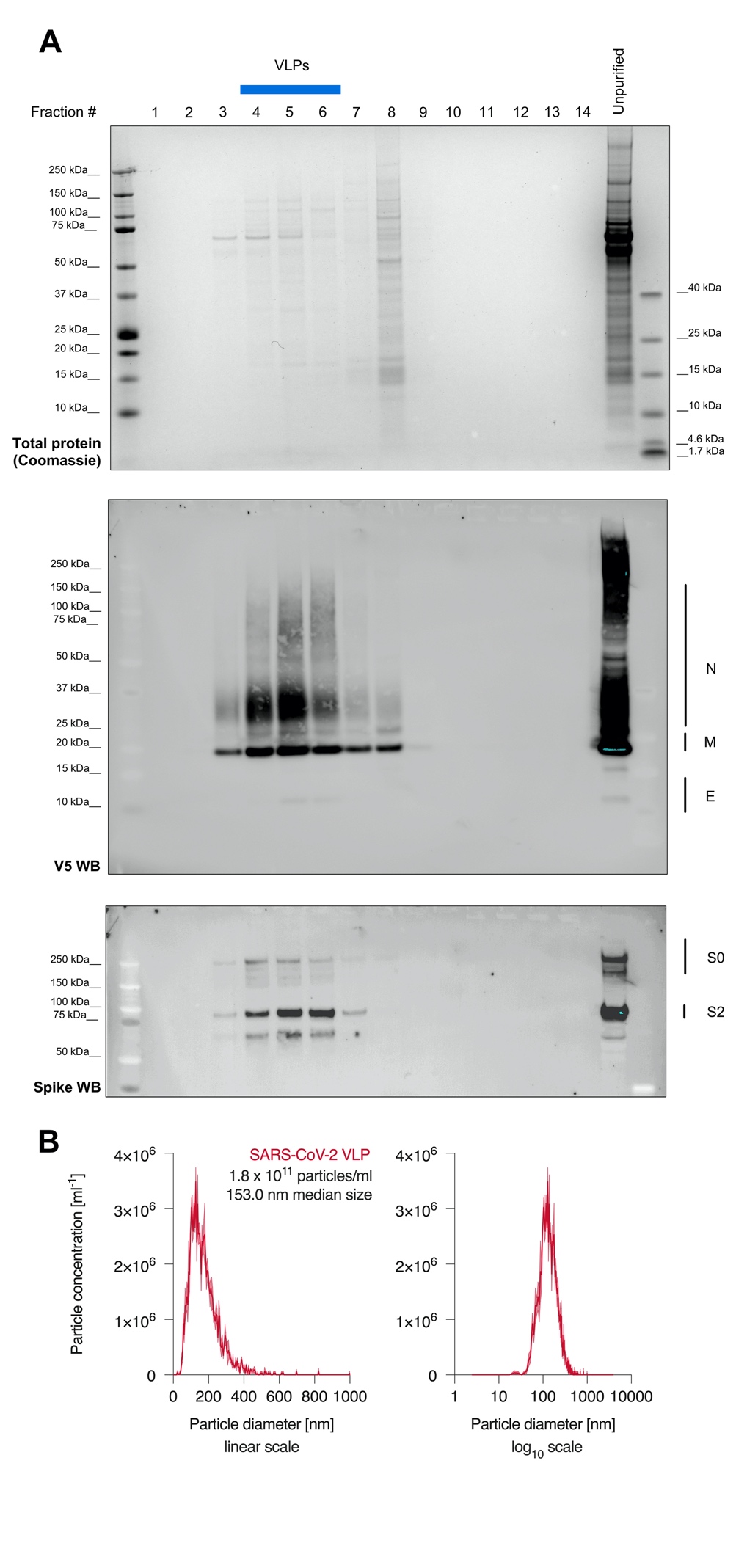


Figure 6S: (A) Preparation of purified SARS-CoV-2 VLPs. SARS-CoV-2-VLPs were prepared as described and purified by OptiPrep gradient ultracentrifugation. Fractions were analyzed for presence of viral strucutral proteins by western blot and total protein by colloidal coomassie staining and those fractions containing the peak signal for all (S, M, E, N) structural proteins pooled (labeled VLP). Bands matching expected sizes of viral structural proteins are annotated. (B) Pooled VLPs were then quantified and characterized by nanoparticle tracking analysis.

**References**

1. Pettersen, E.F.; Goddard, T.D.; Huang, C.C.; Couch, G.S.; Greenblatt, D.M.; Meng, E.C.; Ferrin, T.E. UCSF Chimera - A Visualization System for Exploratory Research and Analysis. *J Comput Chem* **2004**, *25*, 1605–1612.

2. Morris, G.M.; Huey, R.; Lindstrom, W.; Sanner, M.F.; Belew, R.K.; Goodsell, D.S.; Olson, A.J. AutoDock4 and AutoDockTools4: Automated Docking with Selective Receptor Flexibility. *J Comput Chem* **2009**, *30*, 2785–2791.

3. BIOVIA Discovery Studio Visualizer - Dassault Systèmes Available online: https://discover.3ds.com/discovery-studio-visualizer-download (accessed on 5 July 2021).

4. Rajpal, S.; Mizaikoff, B. An in Silico Predictive Method to Select Multi-Monomer Combinations for Peptide Imprinting. *J Mater Chem B* **2022**, *10*, 6618–6626.

5. Batista, A.D.; Rajpal, S.; Keitel, B.; Dietl, S.; Fresco-Cala, B.; Dinc, M.; Groß, R.; Sobek, H.; Münch, J.; Mizaikoff, B. Plastic Antibodies Mimicking the ACE2 Receptor for Selective Binding of SARS-CoV-2 Spike. *Adv Mater Interfaces* **2022**, *9*, 2101925.

6. Udenfriend, S.; Stein, S.; Böhlen, P.; Dairman, W.; Leimgruber, W.; Weigele, M. Fluorescamine: A Reagent for Assay of Amino Acids, Peptides, Proteins, and Primary Amines in the Picomole Range. *Science* **1972**, *178*, 871–872.

7. Xu, R.; Shi, M.; Li, J.; Song, P.; Li, N. Construction of SARS-CoV-2 Virus-Like Particles by Mammalian Expression System. *Front Bioeng Biotechnol* **2020**, *8*, 564639.

8. Hayn, M.; Hirschenberger, M.; Imhof, A.; Kirchhoff, F.; Maria, K.; Correspondence, J.S.; Koepke, L.; Nchioua, R.; Straub, J.H.; Klute, S.; et al. Systematic Functional Analysis of SARS-CoV-2 Proteins Uncovers Viral Innate Immune Antagonists and Remaining Vulnerabilities. *Cell Rep* **2021**, *35*, 109126.

9. Conzelmann, C.; Groß, R.; Zou, M.; Krüger, F.; Görgens, A.; Gustafsson, M.O.; El Andaloussi, S.; Münch, J.; Müller, J.A. Salivary Extracellular Vesicles Inhibit Zika Virus but Not SARS-CoV-2 Infection. *J Extracell Vesicles* **2020**, *9*, 1808281.
